# Detection of Genomically Aberrant Cells within Circulating Tumor Microemboli (CTMs) Isolated from Early-Stage Breast Cancer Patients

**DOI:** 10.3390/cancers13061409

**Published:** 2021-03-19

**Authors:** Marco Silvestri, Carolina Reduzzi, Giancarlo Feliciello, Marta Vismara, Thomas Schamberger, Cäcilia Köstler, Rosita Motta, Stefano Calza, Cristina Ferraris, Andrea Vingiani, Giancarlo Pruneri, Maria Grazia Daidone, Christoph A. Klein, Bernhard Polzer, Vera Cappelletti

**Affiliations:** 1 Biomarker Unit, Department of Applied Research and Technological Development, Fondazione IRCCS Istituto Nazionale dei Tumori di Milano, Via Giovanni Antonio Amadeo 42, 20133 Milano, Italy; marco.silvestri@istitutotumori.mi.it (M.S.); carolina.reduzzi@northwestern.edu (C.R.); marta.vismara@istitutotumori.mi.it (M.V.); rosita.motta@istitutotumori.mi.it (R.M.); mariagrazia.daidone@istitutotumori.mi.it (M.G.D.); 2 Division Personalized Tumor Therapy, Fraunhofer-Institute for Toxicology and Experimental Medicine, Biopark 1|Am Biopark 9, 93053 Regensburg, Germany; giancarlo.feliciello@item.fraunhofer.de (G.F.); caecilia.koestler@item.fraunhofer.de (C.K.); christoph.klein@ukr.de (C.A.K.); bernhard.michael.polzer@item.fraunhofer.de (B.P.); 3 Experimental Medicine and Therapy Research, University Regensburg, Franz-Josef-Strauss Allee 11, 93040 Regensburg, Germany; thomas.schamberger@ukr.de; 4 Unit of Biostatistics, Department of Molecular and Translational Medicine, University of Brescia, Viale Europa 11, 25125 Brescia, Italy; stefano.calza@unibs.it; 5 Department of Medical Epidemiology and Biostatistics, Karolinska Institutet, 171 77 Stockholm, Sweden; 6 Breast Unit, Fondazione IRCCS Istituto Nazionale Dei Tumori di Milano, Via Venezian 1, 20133 Milano, Italy; cristina.ferraris@istitutotumori.mi.it; 7 Department of Pathology and Laboratory Medicine, Fondazione IRCCS Istituto Nazionale dei Tumori di Milano, Via Giacomo Venezian 1, 20133 Milan, Italy; andrea.vingiani@istitutotumori.mi.it (A.V.); giancarlo.pruneri@istitutotumori.mi.it (G.P.); 8 Oncology and Hemato-Oncology Department, University of Milan, Via Festa del Perdono 7, 20122 Milano, Italy

**Keywords:** breast cancer, circulating tumor microemboli, metastatic dissemination, tumor fraction, copy number alteration, low-pass whole genome sequencing

## Abstract

**Simple Summary:**

Distant metastases derive from the shedding and dissemination of single cancer cells (CTCs) or circulating tumor emboli (CTMs) into circulation. Previous studies on CTMs were mainly run in patients with metastatic disease; however, we observed that CTMs are more frequently detected in patients with early-stage breast cancer. Here, we collected single CTMs and their relative primary tumor tissue samples in early-stage patients. By studying genomic aberrations, present in tumors cells and absent in normal cells, we predicted the tumor fraction thanks to a statistical model developed from a calibration curve with breast cancer cell lines. The tumor fraction ranged from 8% to 48% and CTMs contained specific and shared alterations with respect to tissue. Thus, CTMs may derive from different regions of the primary tumor or from occult micrometastases. Moreover, CTM-private mutations may inform us about specific metastasis-associated functions of involved genes that should be further explored in follow-up and mechanistic studies.

**Abstract:**

Circulating tumor microemboli (CTMs) are clusters of cancer cells detached from solid tumors, whose study can reveal mechanisms underlying metastatization. As they frequently comprise unknown fractions of leukocytes, the analysis of copy number alterations (CNAs) is challenging. To address this, we titrated known numbers of leukocytes into cancer cells (MDA-MB-453 and MDA-MB-36, displaying high and low DNA content, respectively) generating tumor fractions from 0–100%. After low-pass sequencing, ichorCNA was identified as the best algorithm to build a linear mixed regression model for tumor fraction (TF) prediction. We then isolated 53 CTMs from blood samples of six early-stage breast cancer patients and predicted the TF of all clusters. We found that all clusters harbor cancer cells between 8 and 48%. Furthermore, by comparing the identified CNAs of CTMs with their matched primary tumors, we noted that only 31–71% of aberrations were shared. Surprisingly, CTM-private alterations were abundant (30–63%), whereas primary tumor-private alterations were rare (4–12%). This either indicates that CTMs are disseminated from further progressed regions of the primary tumor or stem from cancer cells already colonizing distant sites. In both cases, CTM-private mutations may inform us about specific metastasis-associated functions of involved genes that should be explored in follow-up and mechanistic studies.

## 1. Introduction

Despite considerable progress in early diagnosis as well as in the loco-regional and systemic treatment of cancer, the outgrowth of distant metastasis remains responsible for the majority of breast cancer-related deaths [1,2]. Our knowledge on the biology of tumor cell dissemination and metastatic outgrowth is still limited, and molecular key events fundamental for the process need to be identified. These would be valuable as diagnostic tools to predict metastatic spread and as therapeutic targets for personalized treatment strategies.

In patients with solid tumors, hematogenous dissemination plays a major role in the onset of metastatic disease. Circulating tumor cells (CTCs) released from the primary tumor into the bloodstream during the course of the disease have been considered for a long time to be the culprits for metastatic dissemination [3]. However, in addition to single CTCs, numerous studies performed in clinical case series have reported the presence of CTC clusters, also known as circulating tumor microemboli (CTMs), in the blood of metastatic breast cancer patients [4]. The presence of CTMs was found to be associated with overall survival (OS) and progression-free survival (PFS) [5,6,7,8,9], and some studies also reported evidence for added prognostic values of CTM quantification in addition to that of single CTCs [10,11]. 

Most studies addressing the clinical role of clusters in breast cancer have been conducted in metastatic patients, applying CTC enrichment and detection based on the expression of epithelial markers. More recently, by employing a direct approach relying on the use of filtration devices that enrich clusters from whole blood based on their size and by adopting cytomorphological criteria for their identification, we reported that CTMs are over three-times more frequent in women with early breast cancer (EBC) than in metastatic patients [12,13]. This biologically intriguing observation is in line with the hypothesis that dissemination occurs early, allowing the nesting of cancer cells at distant sites a long time before the onset of clinically overt metastases [14,15]. Thus, studying CTMs in EBC may offer an unprecedented chance for both acquiring key knowledge on the initial steps of the metastatic process as well as identifying possible strategies for therapeutic interference.

The remarkably high number of CTMs detected in EBC based on morphological criteria and the variable dimensions of the observed clusters raise questions on their actual cellular composition and in particular on the relative proportion of tumor cells compared to attached non-malignant cells. Evidence for a higher metastatic potential by clusters compared to single CTCs deriving from functional studies has addressed possible mechanisms for this phenomenon by both considering homotypic clusters [16] and underlining the importance of the interaction with accessory cells occurring in heterotypic clusters [17,18]. Different accessory cells have been described to interact with tumor cells within clusters. Among them, neutrophils that interact with tumors cells promoting cell cycle progression [19,20], and myeloid-derived suppressor cells (MDCS) that promote CTC proliferation and immune evasion play a major role [21,22]. 

Here, we hypothesize that characterizing single CTMs at the genomic level will allow us to estimate their cellular composition, evaluate the intra-patient heterogeneity of individual clusters and, in comparison to the primary tumor tissue, highlight subclonal genomic alterations involved in dissemination. Building on a calibration experiment, in which known proportions of breast cancer and blood cells were admixed, we created a weighted mixed regression model for predicting tumor fractions in single CTMs by comparing two algorithms for copy number alteration (CNA) estimation based on low-pass whole genome sequencing (lpWGS). Thereafter, we developed an approach based on the direct isolation of single CTMs by micromanipulation, DNA amplification and CNA profiling by lpWGS and provided evidence, in a small series of EBC patients, that individually isolated CTMs contain tumor cells admixed in variable proportions with accessory non-malignant cells. Finally, by comparing CNAs of CTMs with those of tissue biopsies, we showed that CTMs share genomic aberration with the primary tumor tissue, but also acquire specific alterations.

## 2. Results

### 2.1. Establishment of a Method for Accurate Estimation of Tumor Fraction (TF) in CTMs

To better understand the composition and gain insights into the biological role of CTMs that are frequently detected in EBC [12,13], we developed a model to predict CTMs’ tumor fraction (TF) based on CNA data. First, using a calibration curve of samples formed by breast cancer cell lines admixed with leukocytes, we compared two algorithms for the estimation of tumor genome fraction (TGF) starting from the samples’ CNA profiles. Then, we validated the developed bioinformatic pipeline in tumor samples from EBC patients comparing stroma and tumor fractions. Finally, based on the calibration curve data, we built the statistical model for TF prediction.

#### 2.1.1. Building of a Calibration Curve for Tumor Genome Fraction (TGF) Estimation

For an objective assessment of TGF, defined as a tumor fraction value depending on the relative amount of genomic aberrations in relation to the complete human genome, we have compared the control-FREEC and the ichorCNA algorithms, which to the best of our knowledge are the only ones that compute TGF using CNA data obtained from lp-WGS. 

In order to assess the sensitivity and specificity of ichorCNA and control-FREEC in establishing TGF in mixtures of tumor and normal cells, artificially-generated mixed samples with a known proportion of two cell types were created. For this purpose, we have chosen two breast cancer cell lines characterized by distinct grades of hyperdiploidy and genomic aberrations, MDA-MB-361 (copy average of 2.65) and MDA-MB-453 (copy average of 4.2), and generated cell mixtures containing cancer cells at different ratios to peripheral blood lymphocytes (PBL), ranging from 10% to 100% (pure population considered as benchmark control for aberration calls). Raw sequencing data have been analyzed for quality and compared with the two different bioinformatic pipelines. 

All titration curve replicates passed the pre-alignment and the post-alignment quality control. The median number of aligned reads per sample was 802,875 (range 415,519–1,955,726), with a median samples coverage and median samples mapping quality of 0.04 (range 0.02–0.09) and 51.33 (range 51.98–51.55), respectively. TGF computed by control-FREEC ranged from 0 to 100% with a median value of 78%, whereas the same measurement performed by ichorCNA ranged from 0% to 98.7% with a median of 44.7% (Table S1). Such data suggest a possible overestimation of TGF values by control-FREEC, as discussed in the following section. 

#### 2.1.2. Comparison between Control-FREEC and ichorCNA for TGF Estimation

**Figure 1:**
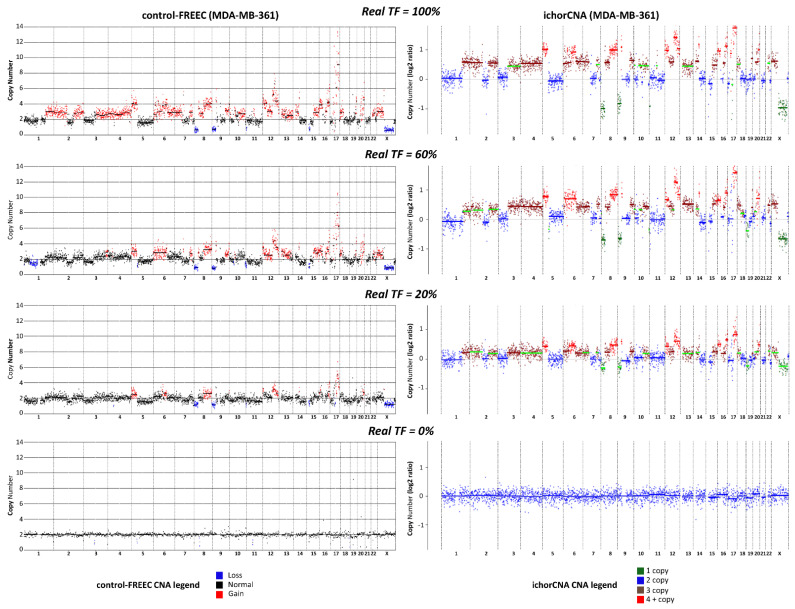
Comparison between ichorCNA and control-FREEC. Copy number alteration (CNA) profiles obtained with low-pass whole genome sequencing (lpWGS) from samples formed by 100% MDA-MB-361 cells (top row), 100% peripheral blood lymphocytes (PBL, bottom row), and artificially-generated mixed samples containing 60% and 20% MDA-MB-361 cells mixed with PBL (second and third row from the top, respectively). CNA profiles reported on the left-hand side were obtained with control-FREEC algorithm; CNA profiles on the right hand side were obtained with ichorCNA. In the case of control-FREEC profiles, different colors refer to loss, gain or their absence (normal) in each genomic region; in the case of ichorCNA, color codes refer to 1 copy, 2 copies, 3 copies, more than 4 copies for each single genomic region. Color codes are reported at the bottom of the figure.

The comparative analysis between the two algorithms showed that ichorCNA allows a more reliable assessment of TGF than control-FREEC, with an overall concordance correlation coefficient (CCC) between real TF and the computed TGF values of 88% and 28%, respectively. When the two cell lines were considered separately, CCC values were 87% (ichorCNA) and 10% (control-FREEC) for MDA-MB361, or 86% (ichorCNA) and 11% (control-FREEC) for MDA-MB-453. The distinct performance in TGF assessment by the two methods was maintained within each replicate, with CCC values of 85% (Rep1), 89% (Rep2), 89% (Rep3) and 44% (Rep1), 28% (Rep2), 4% (Rep3) for ichorCNA and control-FREEC, respectively (Table S1). To better illustrate the difference between the two CNA algorithms, we report in Figure 1 a direct comparison of CNA profiles obtained by the two algorithms in the case of some MDA-MB361/PBL mixed samples.

**Figure 2:**
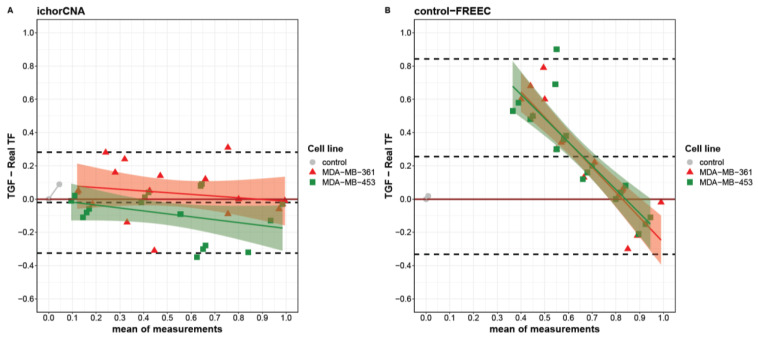
Bland–Altman plot describing the agreement between tumor genome fraction (TGF) measures obtained with ichorCNA (**A**), control-FREEC (**B**) and the real tumor fractions (TFs). Differences between triplicate TGF measures and real TFs are separately reported for MDA-MB-361 (red triangles) and MDA-MB-453 (green squares) as a function of the measures’ averages. Linear fit for each cell line is reported with intervals of confidence. Dashed lines from top to bottom represent the mean of difference plus two standard deviations, mean of difference and mean of difference minus two standard deviations.

When looking at the agreement between real and computed TGF at single points in the titration curve, each algorithm showed a specific behavior. Indeed, using a Bland–Altman plot, we identified different limits of agreement for ichorCNA and control-FREEC ranging from −0.32 to 0.28 and from −0.33 to 0.84, respectively (Figure 2). Globally, a slight underestimation was observed for ichorCNA (−0.02), whereas a significant overestimation was detected for control-FREEC (0.25). Moreover, a clear trend is present in control-FREEC, with a substantial overestimation for low-medium true values and an underestimation for high values (intercept = 0.72). While this trend is also present in ichorCNA, it is much reduced (Figure 2A).

Despite the wide overestimation performed by the control-FREEC algorithm, the TGF assessment by this algorithm was similar between the two cell lines (Figure 2B). A slightly higher divergence was evident between cell lines in ichorCNA with different trends (MDA-MB-361slope = −0.53; MDA-MB-453 slope = −0.52), resulting in an overestimation at real TF = 0 for the MDA-MB-361 and an underestimation at real TF = 1 for MDA-MB-453.

Notably, each method produced an underestimation at real TF = 1, with a median difference of −0.01 and 0.18 for ichorCNA and control-FREEC, respectively. Considering the performance and the better accuracy for the TGF computation by the ichorCNA than the control-FREEC algorithm, ichorCNA was used to analyze tumor and stromal samples collected from patients with EBC. 

#### 2.1.3. Validation of TGF in Stroma and Tumor Fraction from Microdissected Tissues

To further explore the reliability of TGF values in predicting tumor purity, we applied the protocol developed to compute TGF values in the calibration curve experiment to pure tumor samples obtained from six patients with EBC. Patients’ characteristics are reported in Table 1.

To achieve tumor purity, each tumor sample was microdissected to separate stromal from tumor cells.

Briefly, tissue slices were dissociated and cell suspensions were isolated using a digital cell sorting approach [23] based on labeling with DAPI and antibodies against vimentin (VIM) and cytokeratin (CK). Here, we isolated pure fractions of VIM+/CK−stromal cells (S) and VIM−/CK+ tumor cells (T). As CK+ cells could originate from non-malignant breast tissue as well, DAPI staining was used to estimate DNA ploidy of sorted cell fractions by calculating a DNAIndex. For this, stromal cells of each sample were used for an internal normalization reference for diploid cells and DNAIndex was set to 1. For epithelial cells, we applied DNAIndex to distinguish between pseudodiploid cells (T1, DNAIndex around 1) and hyperdiploid cells (T2, DNA index > 1 and > T1). These two populations could be identified in all but patient 3, where we could only detect hyperdiploid cells. The cells were then sorted according to DNAIndex into two separate reaction tubes for subsequent molecular analysis. Details on yields and quality control scores are reported in Table S3.

After lpWGS, all the isolated cell populations passed the pre-alignment and the post-alignment quality controls, allowing us to perform CNA analysis and TGF estimation by ichorCNA. As expected, stromal samples showed TGF values lower than 1% (median TGF = 0.009). TGF values were instead definitely higher in both the pseudodiploid and hyperdiploid tumor fractions (range 0.24 to 0.85). In particular, in three out of five samples, TGF values were higher in the hyperdiploid than in the pseudodiploid fraction, whereas in the remaining two samples the TGF in the pseudodiploid fraction was higher than in T2 in tumor cells (Table 2). Overall, the high TGF values obtained in the pure tumor fractions and the low values of the stromal fractions support the accuracy of TGF estimation for tumor purity evaluation.

#### 2.1.4. Building of a Statistical Model to Predict TF in Clinical Samples Starting from TGF Values

TGF titration data derived from ichorCNA analysis were used to build a model based on linear regression fitted using the linear mixed model (LMM) in order to predict the TF of aberrant CTMs isolated from clinical samples. As opposed to TGF, TF was defined as tumor fraction value corrected for the different degree of genomic aberration (represented by breast cancer cell lines with different ploidies) and for sequencing coverage. 

For such a purpose, the cell lines used in the calibration curve were set as a mixed variable of the model to mimic the biological condition of breast cancer CTMs and the mean absolute error associated with samples coverage as weights (see Materials and Methods Section 4.7). 

The model was characterized by a slight overestimation at real TF = 0 (intercept = 0.049) and a slope of 0.99. Thus, the statistical model is able to take in and input the TGF values and return the corresponding TF.

### 2.2. Genomic Analysis of CTMs Isolated from Blood of Women with EBC

Next, we applied the technical and bioinformatic pipeline developed above to evaluate the TF in mixed samples to CTMs isolated from the blood of early-stage breast cancer patients.

#### 2.2.1. Predicted TF in CTMs of EBC Patients

**Figure 3:**
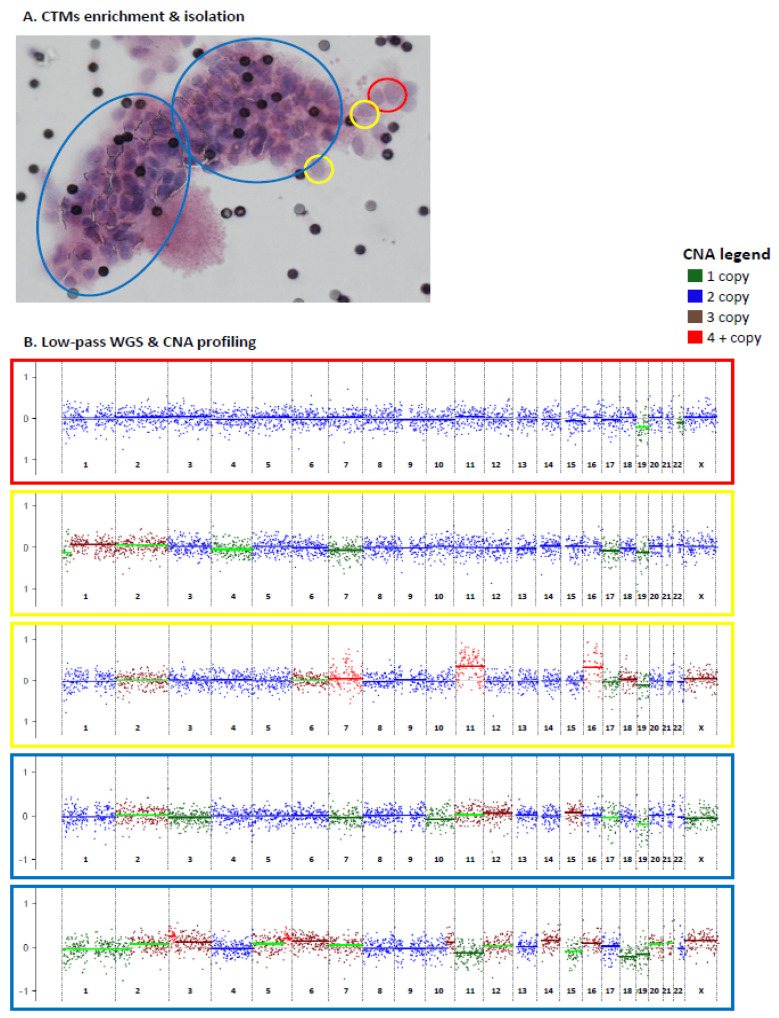
Circulating tumor microemboli (CTMs) isolation and CNA profiling by low-pass whole genome sequencing (lpWGS). Enriched and individually isolated CTMs (either as entire CTMs or divided in parts) (**A**) were subjected to lpWGS for CNA profiling (**B**). Distinct parts of the same CTM are highlighted using different colors (yellow = 1 cell; red = 2 cells, blue = part of CTM. CNA profiles are reported in log-ratio scale between −1 and 1.

The six patients with EBC described above were included in this analysis (Table 1). Blood samples were collected prior to surgery for primary tumors, except for case PT5, whose blood was collected prior to biopsy of a loco-regional relapse occurring following quadrantectomy without any adjuvant systemic treatment. The six blood samples were processed for CTM enrichment by filtration. CTMs, defined as clusters of at least two tumor cells (see Section 4.4 in Material and Methods for details), were individually isolated (either as entire CTMs or divided in parts) by using a micromanipulator. All collected samples, including entire CTMs, parts and single cells isolated from CTMs, were subjected to lpWGS for CNA profiling (Figure 3).

**Figure 4:**
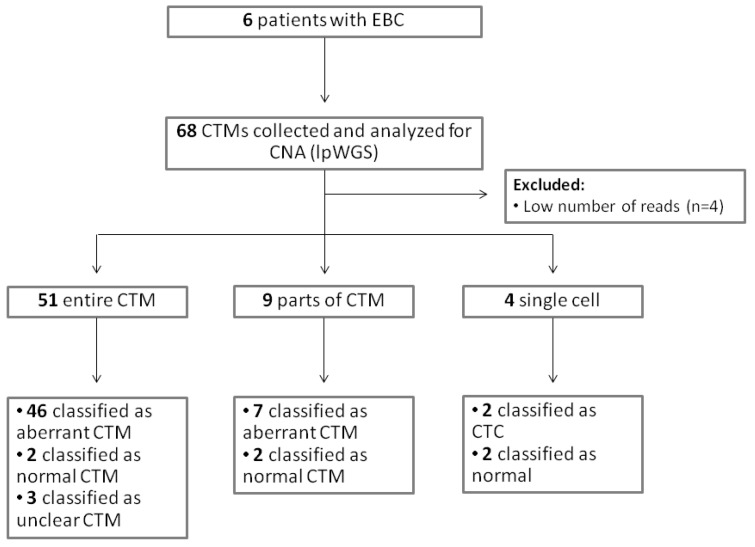
CONSORT for the analyzed samples.

A total of 68 samples (54 entire CTMs, 9 parts of CTMs, and 5 single cells from CTMs) were collected and analyzed for CNA (Table S2). A CONSORT plot for the analyzed samples is reported in Figure 4.

**Figure 5:**
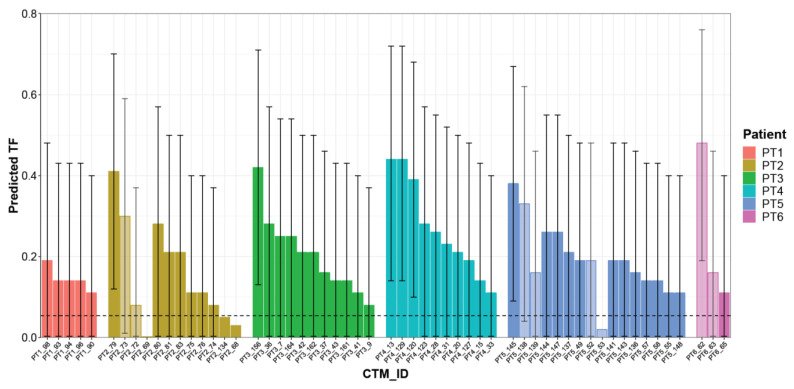
Predicted TF for each CTM. Histogram reporting TF estimated using the mixed logistic regression model for each single CTM. Each color represents a single patient; light-colored bars refer to distinct parts collected from the same CTMs. CTM-IDs are the same as reported in Table S2. Prediction intervals as derived from the linear mixed model (LMM) are reported as error bars.

Considering the 53 CTMs with a confirmed aberrant genome, the LMM model predicted TF values ranging from 0.08 to 0.48 (median 0.21), with a different distribution within each single patient. Patient PT5 had the highest number of aberrant CTMs (14 CTMs, two of them disaggregated into two parts), whereas the lowest numbers of CTMs were detected in patients PT6 and PT1 (2 CTMs, one disaggregated into two parts, and 5 CTMs, respectively). The remaining patients presented comparable numbers of CTMs. For some patients (PT2/PT3/PT4), TF values seem to vary among the collected CTMs, whereas for others (e.g., PT1), all CTMs presented remarkably similar TF values or, as in the case of patient PT5, there were subgroups of CTMs with similar and others with different TF values. Overall, the sample size was too small to draw any conclusion. However, it is interesting to note that in the case of four “splitted CTMs” (PT2, PT5, PT6), different TFs were observed. In general, the CTMs detected in each patient showed a wide level of heterogeneity, suggesting their variable relative proportions of normal and cancer cells (Figure 5 and Table S2).

#### 2.2.2. Comparison of Genomic Alterations of CTMs with Primary Tumor Tissue

In all the patients, TGF values estimated in the two tumor fractions were higher compared to those of the CTMs, confirming the purity of the former ones and the presence of accessory cells in the latter. The two tumor samples (Tumor 1 and Tumor 2) for each patient shared most aberrations as they were strictly correlated despite their distinct DNA indexes. Conversely, the correlations between CTMs from the same patients suggested a high degree of heterogeneity, which is certainly linked to variable TF values (variable presence of normal cells), but also to a variable number of shared alterations (Figure S1).

Next, we focused on altered genomic regions shared among CTMs and tumor tissue from the same patients (Table 3). For all the patients, genomic alterations exclusively detected in the tumor tissue, but not present in any of their CTMs, represented low percentages of the genome (range 1–12%). Indeed, when considering the sum of alterations of all CTMs from a given patient, 31% up to 71% of alterations were shared between CTMs and the tissue. Nonetheless, although to a different degree, the CTMs contained higher percentages of private alterations with respect to the primary tumor. Thus, our data suggest that CTMs are definitely representative of the tumor of origin, but also that they contain peculiar alterations possibly linked with the dissemination process.

**Figure 6:**
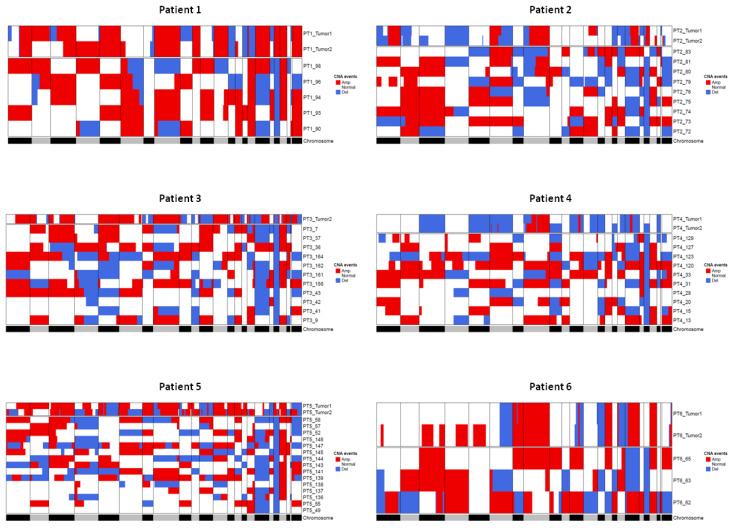
Heatmaps showing comparison between CNAs detected in tumor tissue samples and CTMs from the same patient. Regions of loss are colored blue and regions of gain are colored red. The gray/black lane at the bottom represents the chromosomes from 1 to X and chromosome boundaries are indicated by vertical lines. Tumor 1 and Tumor 2 terms refer to pseudodiploid cells (T1, DNAIndex around 1) and hyperdiploid cells (T2, DNA index > 1 and > T1) population, respectively.

Figure 6 shows for each patient the CNA profile of the tissue and of the corresponding CTMs. CTMs show a remarkable variability in CNA profiles, which can possibly suggest that they originate from distinct regions in the tumor or from occult systemic micrometastatic sites.

A further step was taken to gain some insight into the specific alterations frequently borne by tumors and by CTMs. The top (>70%) shared amplification in tissue samples fell on chromosomes 8, 17 and 20, whereas the top deletions involved chromosomes 11 and 16. In the case of CTMs, the top frequently (>50%) shared amplifications fell on chromosomes 8, 16 and 20, whereas deletions were on chromosome 17 (Table S4). Intriguingly, a search for private alterations of CTMs (i.e., alterations not present on the related tissues) identified numerous alterations on chromosome 2, which deserve further studies (Table S5).

## 3. Discussion

In the current study, we report that CTMs isolated from early-stage breast cancer patients contain genomically aberrant cells and describe the development of a bioinformatic workflow which exploits lpWGS to derive genome-wide copy number profiles and to estimate the tumor fraction (TF). Through the implementation of a weighted-mixed regression model, which relied on the TGF data obtained from an ad hoc calibration experiment to predict the TF, we were able to estimate the TFs of single CTMs isolated in the blood of cancer patients. When applied to 53 aberrant CTMs collected from six women with early breast cancer, our model predicted that 100% of the CTMs contained bona-fide tumor cells with an aberrant DNA, mixed at variable proportions with normal accessory cells. 

Unlike previous studies focused on metastatic breast cancer (MBC), here we collected and molecularly analyzed CTMs from EBC patients, proving their malignant origin. This was made possible by using an innovative model for TF estimation based on CNA data.

The use of lpWGS offers a convenient way to derive genome-wide copy number profiles informing on the presence of cells with aberrant genomes. Indeed, lpWGS of circulating-free DNA (cfDNA) is already a routine procedure for screening fetal anomalies by the detection of large-scale aneuploidy events [24], a procedure that, thanks to its sensitivity, can even lead to the incidental detection of previously unknown maternal malignancies [25,26].

More recently, lpWGS has been used to estimate the tumor fraction of cfDNA isolated from the plasma of patients with advanced tumors including breast and prostate cancer [27,28,29,30], as well as in limited and extensive-stage small cell lung cancer [31]. Thus, bioinformatic tools to perform such a type of extrapolation have already been developed in the field of cfDNA. Indeed, previous studies have shown that, even without prior knowledge of mutations, by using ichorCNA or control-FREEC the tumor fraction can be estimated [28,32]. Here, we exploited these already known bioinformatic tools for a novel application defining a model to estimate the tumor fraction of isolated CTMs.

In our calibration curve experiment using breast cancer cell lines, IchorCNA showed a superior performance with respect to control-FREEC. Its reliability for TGF estimations was further supported by the analysis of stromal and epithelial/tumor cells collected from primary tumors, showing TGF values close to 0 in the case of stromal cells, and ranging from 24% up to 85% for epithelial/tumor cells (including both pseudodiploid and hyperdiploid cells). 

The comparison between control-FREEC and ichorCNA tools in the estimation of TGF value was instrumental for the construction of a prediction model. Taking advantage of the use of two breast cancer cell lines characterized by different degrees of DNA aberration, an LMM model allowed us to predict the TF of CTMs considering our sources of random variability weighted by mean coverage error associated with each point in the titration curve. 

To better understand the model, it is important to remember the difference between TGF and TF as defined in our study. Whereas TGF refers to an estimation of the fraction of tumor DNA only depending on the amount of genomic aberration as computed by control-FREEC or ichorCNA tools, TF is instead defined as a tumor fraction value corrected for the different degree of genomic aberration as modeled using two breast cancer cells lines and for sequencing coverage.

Thus, a possible limitation could be linked to the specific choice of cell lines used for the model. Although we chose cell lines that are representative of breast tumors with different degrees of aberration and of DNA content, the model will change if using different cell lines, and we do not know if it can still be valid when isolating CTMs from patients with other tumor types. Moreover, all reported TF values depend on these two cell lines, and cells with low numbers of aberration may escape detection. Indeed, microdissected tissue samples did not present a 100% TGF value.

Overall, CTMs collected from six patients with early breast cancer showed TF values ranging from 0.08 to 0.48, indicating that accessory cells are widely present in CTMs. The heterotypic nature of CTMs was further supported by the observation that the single cells that were isolated from CTMs showed both aberrant and normal CNAs. 

Having proven that our CTMs do actually contain tumor cells, we asked ourselves to what extent our clusters were representative of the tumor of origin. Notwithstanding the low number of patients, the availability of the primary tumor and the use of a digital microdissection approach [23] that permits the isolation of pure tumor fractions of hyperdiploid and diploid tumor cells allowed us to gain some insight into the genomic comparison between primary tumors and CTMs. 

DNA alterations detected in the two tumor fractions were almost identical, and when compared with the sum of alterations detected in the corresponding CTMs, 30 to 70% of alterations were shared between primaries and CTMs. Thus, tumor cells contained in the clusters are representative of the tumor of origin, but as later discussed, there is a great variability among CTMs from the same patient, suggesting interesting interpretations on their origin. 

Noteworthy, genomic gains and losses that were common to all primaries fell on chromosomes 8, 16 and 17, which have been described in the literature as carriers of the highest CNA burden in breast cancer [33,34]. Chromosome 17 deletions were among the alterations shared in CTMs, in addition to amplification on chromosome 8 and 16. CTMs also shared amplifications on chromosome 20, a long-time known hotspot alteration in breast cancer [35,36].

However, an interesting finding that deserves further studies was that clusters also contained some private alterations, which might help to uncover specific subclones present at a low percentage in the primary tumor, but that can have a role in the dissemination process. Since breast cancer represents a typical DNA copy number-driven tumor [37], it is tempting to speculate that with a larger case series, some interesting suggestions on new mechanisms or candidate genes to be targeted may arise thanks to a systematic study of genome regions altered in CTMs only. It should be noted that the data are also consistent with the interpretation that CTMs stem from hidden metastases where CTM-private alterations had some role in metastatic colonization. Further mechanistic studies are impeded by the fact that within and between individual patients, CTMs do not display uniform patterns of alterations, but are rather heterogeneous. However, as more genomic data on CTM are generated, we may become able to identify candidate genomic regions/genes to be explored in functional studies, and to distinguish them from alterations due to the whole genome amplification (WGA) process.

So far, CTMs have been poorly studied in EBC. Our previous studies [12,13] showed that CTMs are frequently present in the blood of women with stage II and III breast cancer, and now we report that CTMs in M_0_ patients are mostly heterotypic with a variable content of normal cells. Since pre-clinical data from the literature support the idea that heterotypic CTCs have stronger metastatic potential with respect to homotypic clusters or to single CTCs [16], exploring the association between TF and clinical outcome can offer new insight into tumor dissemination and possibly new ways to interfere with it.

## 4. Materials and Methods

### 4.1. Cell Lines and Generation of Cell Mixtures

Breast cancer cell lines MDA-MB-361 (passage number 22) and MDA-MB-453 (passage number 13) [38] were obtained from the American Type Culture Collection (ATCC). Cells were cultured in RPMI (Gibco^TM^ Thermo Fisher Scientific, Waltham, MA, USA) supplemented with 10% fetal calf serum (Gibco^TM^ Thermo Fisher Scientific, Waltham, MA, USA) and 1% penicillin-streptomycin (EuroClone, Pero (MI), Italy)), and maintained at 37 °C and 5% CO_2_. Cell lines were subcultured twice a week at a ratio of 1:2 to 1:6 depending on the confluency. Before isolation, cells were detached using 0.25% trypsin for 5 min. The cells were collected and centrifuged at 4 °C for 5 min and 500× *g*; the pellet was washed once with 1× Phosphate Buffered Saline Solution (PBS), centrifuged again and resuspended in 1–2 mL of 1× PBS [39]

For the generation of cell mixtures that resemble CTMs, cancer cells from MDA-MB-361 and MDA-MB-453 were isolated and admixed together with peripheral blood lymphocytes (PBLs). Single PBLs were collected after centrifugation with Percoll 60% (GE Healthcare) from the blood sample of a healthy female donor after obtaining written consent [40]). Mixtures of 10 cells were generated in order to obtain different ratios of cancer versus normal cells, ranging from 1:10 to 8:10. In addition, a pool of only 10 cancer cells, as well as 10 normal cells, was isolated. Three replicates for each ratio were generated (Table S1). Each time, the exact number of cancer cells and normal cells was transferred to a new field of the chamber slide to ensure the correct titration ratio. The cell mixtures were then isolated by micromanipulation as previously described [41]. Moreover, a negative control consisting of isolation buffer only was generated to exclude any external DNA contamination. 

### 4.2. Case Series

Women with a histologically confirmed diagnosis of stage I–III EBC were recruited at Fondazione IRCCS Istituto Nazionale dei Tumori (INT, Milan, Italy) prior to surgery. Hormone receptor status was evaluated according to the American Society of Clinical Oncology guidelines [42]. HER2 status was considered negative when the immune-histochemical score was 0–1, or 2+ with a negative chromogenic in situ hybridization result [43]. Ki-67 labeling index was assessed by the MIB-1 monoclonal antibody by counting invasive cancer cells at the tumor periphery, without focusing on hot-spots, as recommended by the International Ki-67 in Breast Cancer Working Group [39].

### 4.3. Blood Sample Collection from Breast Cancer Patients

A peripheral blood draw (12 mL) was collected into two 6 mL-K_2_EDTA BD Vacutainer tubes after discarding the first 1–2 mL of blood to avoid contamination by cutaneous cells. Fresh samples were stored at 4 C in the dark and processed within 1 h from withdrawal. All patients provided written informed consent before undergoing any procedures and the CTC/CTM study was approved by the INT Institutional Review Board and Ethics Committee.

### 4.4. CTM Enrichment by a Size-Based Approach

CTM enrichment by size was performed using the ScreenCell^®^ Cyto kit (ScreenCell, Sarcelles France) according to the manufacturer’s instructions, with slight modifications with respect to what was previously described [12,13,14,15,16,17,18,19,20,21,22,23,24,25,26,27,28,29,30,31,32,33,34,35,36,37,38,39,40,41,42,43,44]. Briefly, for each patient, three 3.0 mL aliquots of whole blood were separately mixed with 4 mL of a proprietary red blood cell lysis and fixation buffer (ScreenCell^®^ FC2 filtration buffer) and incubated for 8 min at room temperature. Samples were filtered through three distinct isolation supports (ISs), consisting of a microporous membrane. At the end of the enrichment, ISs were rinsed with PBS, air-dried overnight at room temperature and stained with May Grunwald (Merck Millipore, Burlington, MA, USA). Incubation at room temperature for 2.5 min followed by a second incubation for 2.5 min in May Grunwald diluted 1:2 with water and Giemsa (Merck Millipore; diluted 1:10 with water, 10 min incubation) was performed to allow for the identification of enriched CTMs. CTMs were defined as clusters of at least two cells showing the criteria of malignancy: nuclear size ≥20 μm, nuclear-to-cytoplasmic ratio ≥0.75, irregular nuclear contours and nuclear hyperchromatism. In case the cytoplasm edges were not clearly visible inside the cluster (preventing nuclear-to-cytoplasmic ratio evaluation), malignancy identification was mainly based on nuclei appearance: nuclei scattered irregularly through the cluster and anisokaryosis (i.e., nuclei of variable sizes and shapes), in addition to nuclear size ≥20 μm and irregular nuclear membrane. ISs were stored at 4 °C for 1–2 weeks, until the isolation procedure.

### 4.5. Cell Isolation, Ampli1^TM^ Whole Genome Amplification, DNA Library Construction and Whole Genome Sequencing

Cell mixtures, single cells and CTMs were either isolated from suspension (for generation of cell mixtures for calibration experiments) or from IS using an inverted microscope with micromanipulator (Eppendorf International, Hamburg, Germany). DNA of cell mixtures, single cells and CTMs was amplified using the Ampli1^TM^ WGA kit (Menarini Silicon Biosystems, Castelmaggiore (BO), Italy) based on a published adaptor-ligation-mediated whole genome amplification protocol [45]. The quality of Ampli1^TM^ WGA products was checked as previously described [46], and only products with at least 3 amplified markers were used to prepare sequencing libraries. Five microliters of Ampli1^TM^ WGA product was transferred into a new tube and cleaned up with 1.8X SPRIselect Beads (Beckman Coulter, Brea, CA, USA) according to manufacturer instructions and eluted in 22 μL of nuclease free water. Barcoded libraries for low-pass WGS were prepared either with Ampli1^TM^ LowPass kit for Illumina^®^ platforms or for Ion Torrent^TM^ (Menarini Silicon Biosystems) starting from 10–50 ng of purified Ampli1^TM^ WGA product. The libraries were quantified using Qubit dsDNA HS Assay kit and Qubit 2.0 Fluorometer (Thermo Fisher Scientific, Waltham, MA, USA). Additionally, the average fragment sizes of the libraries were assessed using the Agilent High Sensitivity DNA Kit on the Agilent 2100 Bioanalyzer System (Agilent Technologies, Santa Clara, CA, USA). Sixteen to thirty libraries were pooled in equimolar concentrations to obtain a 4 nM final pool ready for direct sequencing. Ampli1^TM^ LowPass libraries sequencing was performed in single read (SR) mode on a MiSeq System with MiSeq Reagent Kit v3 (150-cycle) (Illumina^®^, San Diego, CA, USA) or on the IonTorrent Ion S5^TM^ system (Thermo Fisher Scientific, Waltham, MA, USA) using the Ion530 chip as per manufacturer’s instructions. 

### 4.6. Isolation of Cancer Cell Populations from Human Formalin-Fixed, Paraffin-Embedded Tissue Sections

Formalin-fixed paraffin-embedded (FFPE) primary tissues from breast cancer patients were dissociated with the DEPArray^TM^ FFPE Sample Prep Kit (Menarini Silicon Biosystems, MSB) and cell number was detected by analyzing 30 µL of sample stained with Hoechst33342. Five hundred thousand cells were stained with a Vimentin/Cytokeratin/DAPI mixture included in the Kit. The quality of DNA for each single sample was determined by analyzing 1500 cells (in triplicates, all presorting measurements performed with Countess^®^ II FL Automated Cell Counter, LifeTech) with the DEPArray^TM^ FFPE QC Kit (MSB), a qPCR based assay using a long and a short primer pair. The ratio between the quantification of the long and the short amplicon, the so called QC score, gives the first DNA quality information about the samples, which should be best higher than 0.2. Eighty thousand cells from each sample were incubated in the DEPArray^TM^ buffer for the recommended time between 16 and 72 h before isolation of cells. Up to 24,000 cells in a final volume of 12 µL were loaded to a DEPArray^TM^ Nxt Cartridge where pictures of each cell were taken for an individual cell selection. From all samples, one population of Vimentin+/Cytokeratin- and one or two populations of Cytokeratin+/Vimentin-cells have been recovered with the DEPArray^TM^ Nxt. The number of cells per population depends on a combination of the QC score and the so called DNAIndex, which is the ratio of DNA content of all cell populations referred to the DNA content of normal, diploid cells in the same sample source (as described in the manufacturer’s manual). It is measured indirectly from the system using the integral intensity of the DAPI signal as a stoichiometric relationship to the cellular DNA content. For the diploid stromal cell population, the DNAIndex is per definition 1 and used as an internal normal reference for DNA content assessment of the cytokeratin positive fraction (see pdf as user manual from Silicon). For Cytokeratin positive cell populations, the DNAIndex can vary from close to one (=near diploid) called pseudo-diploid fraction, whereas cells with a DNAIndex < 1 might be fragmented, necrotic, apoptotic cells but cells with a DNAIndex > 1 and higher than the DNAIndex of the pseudo-diploid cells are the hyper-diploid cells. This information together allows us to calculate the effectively amplifiable template (EAT = QC score × ploidy(=DNAIndex) × number of cells), which has a predictive value for the outcome of library preparation and should be at least 30 for processing the sample with the FFPE LowPass sequencing kit (see previous section). The size of the collected cell population varies as a function of actual population numbers, available cell numbers per population and free parking positions (max. 1000 in total for the FFPE application) in the DEPArray^TM^ Nxt cartridge. 

### 4.7. Sequencing Data Analysis

Illumina raw sequences were checked for quality using fastQC tool (http://www.bioinformatics.babraham.ac.uk/projects/fastqc. accessed on 15 January 2019) (Table S6), aligned to the human reference genome (hg19) with Burrows-Wheeler Aligner (BWA-MEM algorithm) and subjected to Qualimap2 [47] for alignment quality control. 

IonTorrent raw sequences were checked for quality as well as for Illumina samples (Table S6) and aligned to the human reference genome (hg19) with tmap aligner tool using Torrent_Suite 5.10.0. Samples with aligned reads counts lower than 400,000 were excluded from the analysis.

For titration curves, the tumor genome fraction estimations (TGF) were obtained from copy number alteration (CNA) profiles by using control-FREEC [32] and ichorCNA [28] tools with the following settings:

- control-FREEC. coefficientofVariation = 0.05, mateOrientation = 0, normal control = TRUE, window = 1 Mb, ploidy = 2.65 (MDA-MB-361) or 4.2 (MDA-MB-453);
- ichorCNA. Window = 1 Mb, ploidy = 2.65 (MDA-MB-361) or 4.2 (MDA-MB-453), estimatePloidy = FALSE, estimateNormal = TRUE, normalPanel = TRUE, normal state = c(0.1, 0.2, 0.3, 0.4, 0.5, 0.6, 0.7, 0.8, 0.9).

For single, cluster, and tissue cell populations obtained from clinical samples, TGF estimations were obtained using ichorCNA tool with window = 1Mb, ploidy = 2, estimatePloidy = TRUE, estimateNormal = TRUE, normalPanel = TRUE and normal state = c(0.1, 0.2, 0.3, 0.4, 0.5, 0.6, 0.7, 0.8, 0.9). CTMs were finally classified based on criteria reported in the following table (Table 4).

Considering the evaluation of the CNA profile, chr19 was not considered due to its biased deletion associated with the high CG base percentage [48]. Unclear CNA profiles were related to samples that showed one of the following features:

- Normal profile but only 1 genomic region with amplification/deletion lower than 125 Mb;
- Normal profile but sum of amplification/deletion of different genomic regions lower than 375 Mb.

All CNA profiles with alterations above these thresholds were classified as aberrant. None of the normal controls (both single and pool of leukocites) presented an aberrant profile, as already published [49].

### 4.8. Statistical Analysis

The concordance between standard curve real TF values and TGFs estimated by control-FREEC and by ichorCNA tool was assessed using the concordance correlation coefficient (CCC), which quantifies the agreement between two measures [50], and the Bland–Altman method [51]. 

Starting from previously reported ichorCNA mean absolute errors associated with sequencing depth of coverage [28], the specific error associated with each sample in titration curve were compute by linear interpolation. These data were then considered as weights within the fitted Linear Mixed Regression Model [52], setting a random intercept for each cell line and transforming the proportion on logit scale before.

The LMM model was then used to predict TF and related intervals of confidence and prediction of aberrant CTMs derived from clinical samples with an inverse estimation [53]. Patient-specific correlation matrices between the CNA profile of tissue cell populations and CTMs were computed using Pearson’s coefficient and submitted to hierarchical cluster analysis considering Euclidean distance and the Complete linkage method [54].

The shared and private CNA events among tumors and CTMs were derived from CNA segmentation file of each sample. Depending on the different analysis performed, genomic regions were defined as “shared” if characterized by the same type of alteration (amplification/deletion) in one of the following conditions:

- Both tumor samples of the same patients (when possible);
- Both tumor samples and at least one CTM of the same patient;
- At least two CTMs of the same patients.

The “private” label was assigned to CNA events never shared between CTMs and tumor samples of the same patients. Considering the detection of private alterations among the 53 aberrant CTMs, genomic regions classified as “shared” even just in 1 patient or altered in less than 55% of the CTMs were excluded from the analysis. 

Genomic annotations associated with each genomic position were retrieved using considering UCSC genome browser and Ensemble resources and hg19 as reference genome. CancerIndex database was used to identify genes associated with breast cancer.

All the statistical analyses were performed using R software (see Table S6 for the R packages detailed).

## 5. Conclusions

Our study reports a genomic proof for the presence of malignant cells admixed with normal cells within CTMs isolated from six women with early breast cancer by exploiting lpWGS to estimate the TF, thanks to the implementation of a linear mixed model. Moreover, comparing CNA profiles from the corresponding primary tumors with those obtained in CTMs, we show that although they are representative of the tumor of origin, CTMs acquire specific alterations possibly involving genomic regions containing genes involved in dissemination. Moreover, this suggests that CTMs may derive from a different region from the primary tumor with a higher seeding ability or from occult micrometastases.

## Figures and Tables

**Table 1:** Tumor pathological characteristic.

	PT1	PT2	PT3	PT4	PT5	PT6
pT	1.5	2.2	2.5	1.2	/	1.7
Histotype	IDC	IDC	IDC	IDC muc	IC	IDC
Histological grade	G2	G3	G3	G2	G3	G2
pN	N+	N+	N+	N_0_	/	N+
ER	Pos	Neg	Pos	Pos	/	Pos
PgR	Pos	Pos	Pos	Pos	/	Pos
HER2	2+	3+	1+	0	/	1+
Ki67	35%	70%	75%	30%	/	22%

**Table 2:** TGF values of Stromal and Tumor tissue samples for each patient.

Patient	Stromal	Tumor 1 *	Tumor 2 *
PT1	0.01	0.25	0.53
PT 2	0.004	0.24	0.72
PT 3	0.01	NA	0.35
PT 4	0.006	0.66	0.64
PT 5	0.009	0.31	0.53
PT 6	0.01	0.85	0.67

* Tumor 1 and Tumor 2 terms refer to pseudodiploid cells (T1, DNAIndex around 1) and hyperdiploid cells (T2, DNA index > 1 and > T1) population, respectively.

**Table 3:** Private and shared genomic regions between CTMs and Tumor tissue for each patient.

**Patient**	**CNA Private (Tumor)**	**CNA Private (CTM)**	**Sum of CNA Shared**
PT1	0.08	0.52	0.44
PT2	0.04	0.62	0.39
PT3	0.05	0.3	0.71
PT4	0.12	0.63	0.37
PT5	0.01	0.44	0.56
PT6	0.04	0.62	0.31
**Legend**			
CNA Private (Tumor)		Number of CNA exclusive of the tumors/total of genome	
CNA Private (CTM)		Number of CNA exclusive of the CTMs/total of genome	
Sum of CNA shared		Sum of CNA shared between both tumors and at least 1 CTMs/total of genome	

**Table 4:** Private and shared genomic regions between CTMs and Tumor tissue for each patient.

TGF	+	CNA Profile	=	Final Output
0 ≤ TGF ≤ 0.05	+	Normal/Aberrant/Unclear	=	Normal CTM
0.05 < TGF ≤ 1	+	Aberrant	=	Aberrant CTM
0.05 < TGF ≤ 1	+	Unclear	=	Unclear CTM

## Data Availability

The datasets generated during the current study are available from the corresponding author on request.

## Supplementary Materials

The following are available online at https://www.mdpi.com/2072-6694/13/6/1409/s1, Figure S1: Correlation between CNA profile of tumor tissue and CTMs within each patient; Table S1A: Comparison between TGF computation performed by ichorCNA and control-FREEC; Table S1B: ichorCNA fitted coverage error associated with each point of titration curve; Table S2A: TF values and related intervals of prediction returned by LMM model; Table S2B: TF values and related intervals of confidence returned by LMM model; Table S3: Yields and quality control scores related to microdissected primary tumor samples; Table S4A: Top (>70%) shared amplifications between Tumor tissue samples; Table S4B: Top (>70%) shared deletions between Tumor tissues samples; Table S4C: Top (>50%) shared amplifications between CTMs; Table S4D: Top (>50%) shared deletions between CTMs; Table S5A: Genomic regions associated with private alterations of CTMs; Table S5B: Genomic regions associated with private alterations of CTMs involved in Breast cancer (CancerIndex); Table S6A: Quality score associated with cell lines raw sequences; Table S6B: Quality score associated with single and cluster of cells raw sequences; Table S6C: Quality score associated with tissue cell populations raw sequences; Table S7: R packages considered for statistical analysis.
