# Supplementary material for: Detection of Genomically Aberrant Cells within Circulating Tumor Microemboli (CTMs) Isolated from Early-Stage Breast Cancer Patients
Source: Cancers (Basel). 2021 Mar 19;13(6):1409. doi: 10.3390/cancers13061409 (PMC8003526; doi:10.3390/cancers13061409)
Supplement: Supplementary file 1 [file cancers-13-01409-s001.pdf]

cancers

MDPI

Article

Detection of Genomically Aberrant Cells within Circulating Tumor Microemboli (CTMs) Isolated from Early-Stage Breast Cancer Patients

Marco Silvestri [1,†], Carolina Reduzzi [1,†], Giancarlo Feliciello [2], Marta Vismara [1], Thomas Schamberger [3], Cäcilia Köstler[2], Rosita Motta [1], Stefano Calza [4,5], Cristina Ferraris [6], Andrea Vingiani [7,8], Giancarlo Pruneri [7,8], Maria Grazia Daidone [1], Christoph A. Klein [2,3], Bernhard Polzer [2,‡] and Vera Cappelletti [1,*,‡]

1 Biomarker Unit, Department of Applied Research and Technological Development, Fondazione IRCCS Istituto Nazionale dei Tumori di Milano, Via Giovanni Antonio Amadeo 42, 20133 Milano, Italy; marco.silvestri@istitutotumori.mi.it (M.S.); carolina.reduzzi@northwestern.edu (C.R.); marta.vismara@istitutotumori.mi.it (M.V.); rosita.motta@istitutotumori.mi.it (R.M.); mariagrazia.daidone@istitutotumori.mi.it (M.G.D.)
2 Division Personalized Tumor Therapy, Fraunhofer-Institute for Toxicology and Experimental Medicine, Biopark 1 | Am Biopark 9, 93053 Regensburg, Germany; giancarlo.feliciello@item.fraunhofer.de (G.F.); caecilia.koestler@item.fraunhofer.de (C.K.); christoph.klein@ukr.de (C.A.K.); bernhard.michael.polzer@item.fraunhofer.de (B.P.)
3 Experimental Medicine and Therapy Research, University Regensburg, Franz-Josef-Strauss Allee 11, 93040 Regensburg, Germany; thomas.schamberger@ukr.de
4 Unit of Biostatistics, Department of Molecular and Translational Medicine, University of Brescia, Viale Europa 11, 25125 Brescia, Italy; stefano.calza@unibs.it
5 Department of Medical Epidemiology and Biostatistics, Karolinska Institutet, 171 77 Stockholm, Sweden
6 Breast Unit, Fondazione IRCCS Istituto Nazionale Dei Tumori di Milano, Via Venezian 1, 20133 Milano, Italy; cristina.ferraris@istitutotumori.mi.it
7 Department of Pathology and Laboratory Medicine, Fondazione IRCCS Istituto Nazionale dei Tumori di Milano, Via Giacomo Venezian 1, 20133 Milan, Italy; andrea.vingiani@istitutotumori.mi.it (A.V.); giancarlo.pruneri@istitutotumori.mi.it (G.P.)
8 Oncology and Hemato-Oncology Department, University of Milan, Via Festa del Perdono 7, 20122 Milano, Italy
* Correspondence: vera.cappelletti@istitutotumori.mi.it
† Equal contribution M.S. and C.R. are co-first authors.
‡ Equal contribution B.P. and V.C. are co-last authors.

Supplementary

Citation: Silvestri, M.; Reduzzi, C.; Feliciello, G.; Vismara, M.; Schamberger, T.; Koestler, C.; Motta, R.; Calza, S.; Ferraris, C.; Vingiani, A.; et al. Detection of Genomically Aberrant Cells within Circulating Tumor Microemboli (CTMs) Isolated from Early-Stage Breast Cancer Patients. *Cancers* **2021**, *13*, 1409. https://doi.org/10.3390/cancers13061409

Academic Editor: Fabio Puglisi

Received: 28 January 2021
Accepted: 15 March 2021
Published: 19 March 2021

Publisher's Note: MDPI stays neutral with regard to jurisdictional claims in published maps and institutional affiliations.

Copyright: © 2021 by the author. Licensee MDPI, Basel, Switzerland. This article is an open access article distributed under the terms and conditions of the Creative Commons Attribution (CC BY) license (http://creativecommons.org/licenses/by/4.0/).

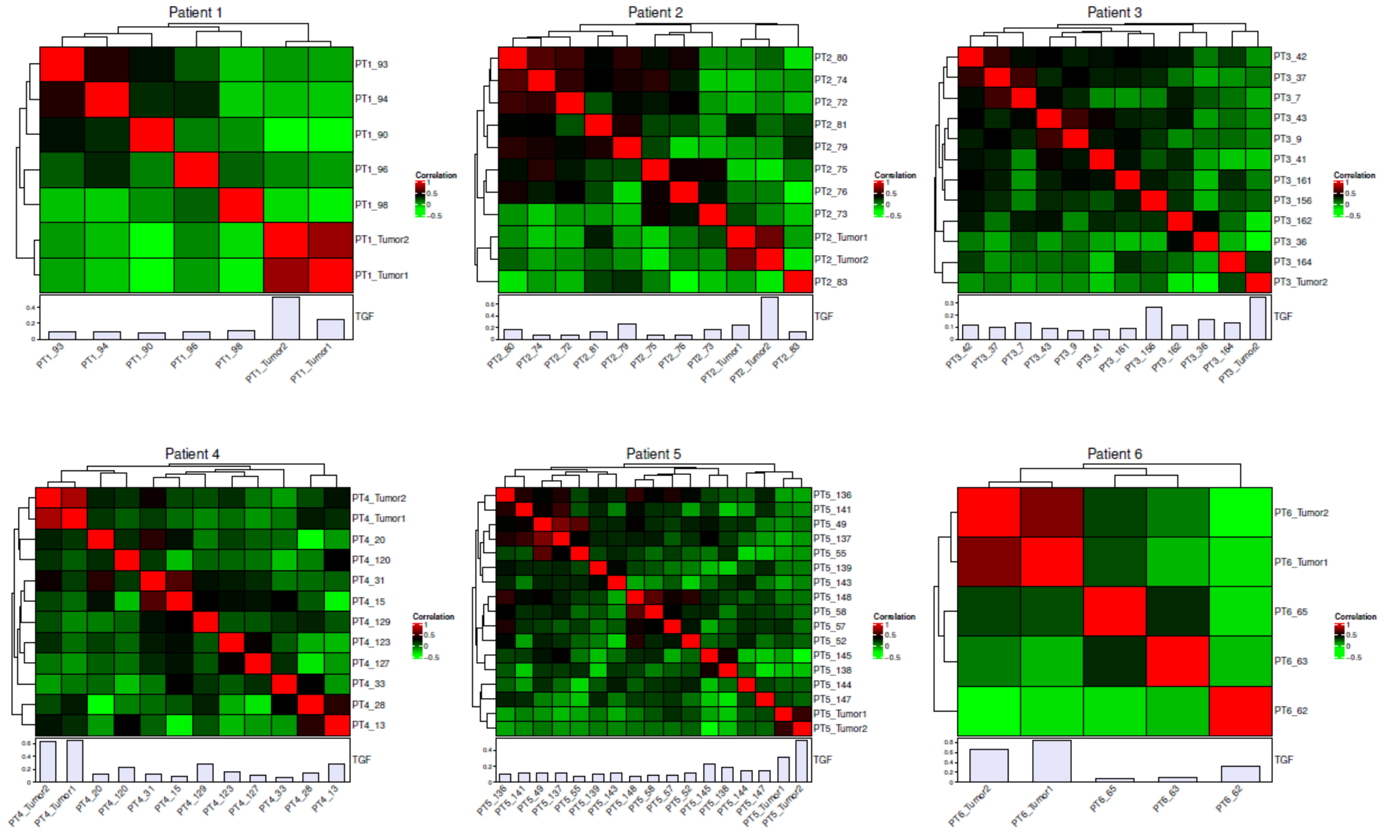

Figure S1. Correlation between CNA profile of tumor tissue and CTMs within each patient.

Table S1A. Comparison between TGF computation performed by ichorCNA and control-FREEC.

Cell lines	Tumor_fraction expected	Replicates	Tumor_Genome_Fraction_ichorCNA	Tumor_Genome_Fraction_control-FREEC
**MDA-MB-361**	10%	1	14.14%	78.35%
		2	15.35%	89.22%
		3	37.83%	70.23%
	20%	1	35.70%	68.11%
		2	43.73%	70.48%
		3	17.08%	80.27%
	40%	1	45.10%	74.73%
		2	25.60%	74.39%
		3	53.56%	69.53%
	60%	1	28.72%	73.41%
		2	91.00%	81.55%
		3	71.85%	82.02%
	80%	1	71.40%	81.98%
		2	70.69%	84.97%
		3	79.63%	85.86%
	100%	1	98.76%	98.19%
		2	96.85%	69.55%
		3	94.05%	77.95%
**MDA-MB-453**	10%	1	12.23%	68.08%
		2	8.53%	62.58%
		3	11.89%	100.00%
	20%	1	14.24%	70.25%
		2	9.11%	89.28%
		3	11.71%	67.89%
	40%	1	44.41%	78.03%
		2	41.05%	76.25%
		3	37.90%	70.41%

	60%	1	68.71%	76.35%
		2	68.01%	79.71%
		3	50.76%	71.65%
	80%	1	44.98%	84.08%
		2	50.08%	80.09%
		3	52.25%	88.33%
	100%	1	68.18%	88.65%
		2	97.08%	85.22%
		3	86.66%	79.49%
**Control**	0%	1	0.00%	0.00%
		2	8.72%	2.00%

Table S1B. ichorCNA fitted coverage error associated with each point of titration curve.

Cell lines	Tumor_fraction expected	Replicates	Tumor_Genome_Fraction_ichorCNA	Mean_coverage	Fitted_Coverage_Error
**MDA-MB-361**	10%	1	14.14%	0.0387	0.0217777049180328
		2	15.35%	0.0505	0.0200754098360656
		3	37.83%	0.029	0.0231770491803279
	20%	1	35.70%	0.0584	0.018935737704918
		2	43.73%	0.0497	0.0201908196721312
		3	17.08%	0.0595	0.0187770491803279
	40%	1	45.10%	0.0554	0.0193685245901639
		2	25.60%	0.0496	0.0202052459016394
		3	53.56%	0.0445	0.0209409836065574
	60%	1	28.72%	0.0413	0.0214026229508197
		2	91.00%	0.0365	0.0220950819672131
		3	71.85%	0.0607	0.0186039344262295
	80%	1	71.40%	0.0555	0.0193540983606557
		2	70.69%	0.0938	0.0138288524590164
		3	79.63%	0.0607	0.0186039344262295
	100%	1	98.76%	0.0381	0.021864262295082
		2	96.85%	0.0318	0.0227731147540984
		3	94.05%	0.0385	0.0218065573770492
**MDA-MB-453**	10%	1	12.23%	0.0312	0.0228596721311475
		2	8.53%	0.0273	0.0234222950819672
		3	11.89%	0.0246	0.0238118032786885
	20%	1	14.24%	0.0323	0.0227009836065574
		2	9.11%	0.0257	0.0236531147540984
		3	11.71%	0.0365	0.0220950819672131
	40%	1	44.41%	0.0323	0.0227009836065574
		2	41.05%	0.0204	0.0244177049180328
		3	37.90%	0.0215	0.0242590163934426
	60%	1	68.71%	0.0201	0.0244609836065574
		2	68.01%	0.0502	0.0201186885245902
		3	50.76%	0.0495	0.0202196721311475
	80%	1	44.98%	0.0368	0.0220518032786885
		2	50.08%	0.027	0.0234655737704918
		3	52.25%	0.0558	0.0193108196721311
	100%	1	68.18%	0.0292	0.0231481967213115
		2	97.08%	0.0512	0.0199744262295082
		3	86.66%	0.0559	0.019296393442623
**Control**	0%	1	0.00%	0.0262	0.0235809836065574
		2	8.72%	0.0197	0.0245186885245902

Table S2A. TF values and related intervals of prediction returned by LMM model.

Patient	CTM_ID	Sequencing_Platform	CTC_CTM	CTM_type	GII	Call	ichorCNA_TGF	Predicted_TF	lwr	upr
PT1	PT1_90	IonTorrent_S5XL	CTM	Whole	4	aberrant	0.08	0.113	0	0.402
	PT1_93	Illumina_MiSeq	CTM	Whole	4	aberrant	0.09	0.14	0	0.43
	PT1_94	Illumina_MiSeq	CTM	Whole	4	aberrant	0.09	0.14	0	0.43
	PT1_96	Illumina_MiSeq	CTM	Whole	4	aberrant	0.09	0.14	0	0.43
	PT1_98	Illumina_MiSeq	CTM	Whole	4	aberrant	0.11	0.188	0	0.478

PT2	PT2_68	IonTorrent_S5XL	CTM	Whole	4	normal	0.03			
	PT2_72	IonTorrent_S5XL	CTM	Divided	4	aberrant	0.07	0.082	0	0.371
	PT2_73	IonTorrent_S5XL	CTM	Divided	4	aberrant	0.17	0.297	0.007	0.586
	PT2_69	IonTorrent_S5XL	CTM	Divided	4	normal	0			
	PT2_74	IonTorrent_S5XL	CTM	Whole	4	aberrant	0.07	0.082	0	0.371
	PT2_75	IonTorrent_S5XL	CTM	Whole	4	aberrant	0.08	0.113	0	0.402
	PT2_76	IonTorrent_S5XL	CTM	Whole	4	aberrant	0.08	0.113	0	0.402
	PT2_77	IonTorrent_S5XL	CTM	Whole	4	low_reads_count				
	PT2_79	IonTorrent_S5XL	CTM	Whole	3	aberrant	0.26	0.413	0.123	0.702
	PT2_80	IonTorrent_S5XL	CTM	Whole	4	aberrant	0.16	0.281	0	0.571
	PT2_81	IonTorrent_S5XL	CTM	Whole	3	aberrant	0.12	0.209	0	0.499
	PT2_83	IonTorrent_S5XL	CTM	Whole	3	aberrant	0.12	0.209	0	0.499
	PT2_131	IonTorrent_S5XL	CTM	Whole	4	low_reads_count				
	PT2_133	IonTorrent_S5XL	CTM	Whole	4	low_reads_count				
	PT2_134	IonTorrent_S5XL	CTM	Whole	4	unclear	0.05			
	PT2_70	IonTorrent_S5XL	CTC			Aberrant				
	PT2_71	IonTorrent_S5XL	CTC			Normal				
PT3	PT3_7	Illumina_MiSeq	CTM	Whole	3	aberrant	0.14	0.248	0	0.537
	PT3_9	IonTorrent_S5XL	CTM	Whole	4	aberrant	0.07	0.082	0	0.371
	PT3_36	Illumina_MiSeq	CTM	Whole	4	aberrant	0.16	0.281	0	0.571
	PT3_37	Illumina_MiSeq	CTM	Whole	4	aberrant	0.1	0.165	0	0.455
	PT3_41	IonTorrent_S5XL	CTM	Whole	4	aberrant	0.08	0.113	0	0.402
	PT3_42	IonTorrent_S5XL	CTM	Whole	4	aberrant	0.12	0.209	0	0.499
	PT3_43	IonTorrent_S5XL	CTM	Whole	4	aberrant	0.09	0.14	0	0.43
	PT3_156	Illumina_MiSeq	CTM	Whole	4	aberrant	0.27	0.424	0.134	0.713
	PT3_161	Illumina_MiSeq	CTM	Whole	4	aberrant	0.09	0.14	0	0.43
	PT3_162	Illumina_MiSeq	CTM	Whole	4	aberrant	0.12	0.209	0	0.499
	PT3_164	Illumina_MiSeq	CTM	Whole	4	aberrant	0.14	0.248	0	0.537
	PT3_44	IonTorrent_S5XL	CTC			aberrant				
PT4	PT4_13	IonTorrent_S5XL	CTM	Whole	4	aberrant	0.28	0.435	0.145	0.724
	PT4_15	IonTorrent_S5XL	CTM	Whole	4	aberrant	0.09	0.14	0	0.43
	PT4_20	IonTorrent_S5XL	CTM	Whole	4	aberrant	0.12	0.209	0	0.499
	PT4_27	IonTorrent_S5XL	CTM	Whole	4	unclear	0.2			
	PT4_28	IonTorrent_S5XL	CTM	Whole	4	aberrant	0.15	0.265	0	0.554
	PT4_31	IonTorrent_S5XL	CTM	Whole	4	aberrant	0.13	0.229	0	0.519
	PT4_33	IonTorrent_S5XL	CTM	Whole	4	aberrant	0.08	0.113	0	0.402
	PT4_118	Illumina_MiSeq	CTM	Whole	4	unclear	0.09			
	PT4_120	Illumina_MiSeq	CTM	Whole	4	aberrant	0.24	0.39	0.1	0.679
	PT4_123	Illumina_MiSeq	CTM	Whole	4	aberrant	0.16	0.281	0	0.571
	PT4_125	Illumina_MiSeq	CTM	Whole	4	unclear	0.22			
	PT4_127	Illumina_MiSeq	CTM	Whole	4	aberrant	0.11	0.188	0	0.478
	PT4_129	Illumina_MiSeq	CTM	Whole	4	aberrant	0.28	0.435	0.145	0.724
PT5	PT5_49	IonTorrent_S5XL	CTM	Whole	4	aberrant	0.11	0.188	0	0.478
	PT5_52	Illumina_MiSeq	CTM	Divided	4	aberrant	0.11	0.188	0	0.478
	PT5_53	IonTorrent_S5XL	CTM	Divided	4	normal	0.02			
	PT5_55	IonTorrent_S5XL	CTM	Whole	4	aberrant	0.08	0.113	0	0.402
	PT5_57	Illumina_MiSeq	CTM	Whole	4	aberrant	0.09	0.14	0	0.43
	PT5_58	Illumina_MiSeq	CTM	Whole	4	aberrant	0.09	0.14	0	0.43
	PT5_136	Illumina_MiSeq	CTM	Whole	4	aberrant	0.1	0.165	0	0.455
	PT5_137	Illumina_MiSeq	CTM	Whole	4	aberrant	0.12	0.209	0	0.499
	PT5_138	Illumina_MiSeq	CTM	Divided	3	aberrant	0.19	0.326	0.037	0.616
	PT5_139	Illumina_MiSeq	CTM	Divided	4	aberrant	0.1	0.165	0	0.455
	PT5_141	Illumina_MiSeq	CTM	Whole	3	aberrant	0.11	0.188	0	0.478
	PT5_143	Illumina_MiSeq	CTM	Whole	3	aberrant	0.11	0.188	0	0.478
	PT5_144	Illumina_MiSeq	CTM	Whole	4	aberrant	0.15	0.265	0	0.554
	PT5_145	Illumina_MiSeq	CTM	Whole	4	aberrant	0.23	0.378	0.088	0.668
	PT5_147	Illumina_MiSeq	CTM	Whole	3	aberrant	0.15	0.265	0	0.554
	PT5_148	Illumina_MiSeq	CTM	Whole	3	aberrant	0.08	0.113	0	0.402
	PT5_54	IonTorrent_S5XL	CTC			Normal				
	PT5_56	IonTorrent_S5XL	CTC			low_reads_count				
PT6	PT6_62	Illumina_MiSeq	CTM	Divided	4	aberrant	0.32	0.476	0.186	0.765
	PT6_63	Illumina_MiSeq	CTM	Divided	4	aberrant	0.1	0.165	0	0.455
	PT6_65	Illumina_MiSeq	CTM	Whole	4	aberrant	0.08	0.113	0	0.402

Table S2B. TF values and related intervals of confidence returned by LMM model.

Patient	CTM_ID	Sequencing_Platform	CTC_CTM	CTM_type	GII	Call	ichorCNA_TGF	Predicted_TF	lwr	upr
PT1	PT1_90	IonTorrent_S5XL	CTM	Whole	4	aberrant	0.08	0.113	0	0.418
	PT1_93	Illumina_MiSeq	CTM	Whole	4	aberrant	0.09	0.14	0	0.446
	PT1_94	Illumina_MiSeq	CTM	Whole	4	aberrant	0.09	0.14	0	0.446
	PT1_96	Illumina_MiSeq	CTM	Whole	4	aberrant	0.09	0.14	0	0.446
	PT1_98	Illumina_MiSeq	CTM	Whole	4	aberrant	0.11	0.188	0	0.494
PT2	PT2_68	IonTorrent_S5XL	CTM	Whole	4	normal	0.03			
	PT2_72	IonTorrent_S5XL	CTM	Divided	4	aberrant	0.07	0.082	0	0.386
	PT2_73	IonTorrent_S5XL	CTM	Divided	4	aberrant	0.17	0.297	0	0.606
	PT2_69	IonTorrent_S5XL	CTM	Divided	4	normal	0			
	PT2_74	IonTorrent_S5XL	CTM	Whole	4	aberrant	0.07	0.082	0	0.386
	PT2_75	IonTorrent_S5XL	CTM	Whole	4	aberrant	0.08	0.113	0	0.418
	PT2_76	IonTorrent_S5XL	CTM	Whole	4	aberrant	0.08	0.113	0	0.418
	PT2_77	IonTorrent_S5XL	CTM	Whole	4	low_reads_count				
	PT2_79	IonTorrent_S5XL	CTM	Whole	3	aberrant	0.26	0.413	0.103	0.728
	PT2_80	IonTorrent_S5XL	CTM	Whole	4	aberrant	0.16	0.281	0	0.59
	PT2_81	IonTorrent_S5XL	CTM	Whole	3	aberrant	0.12	0.209	0	0.516
	PT2_83	IonTorrent_S5XL	CTM	Whole	3	aberrant	0.12	0.209	0	0.516
	PT2_131	IonTorrent_S5XL	CTM	Whole	4	low_reads_count				
	PT2_133	IonTorrent_S5XL	CTM	Whole	4	low_reads_count				
	PT2_134	IonTorrent_S5XL	CTM	Whole	4	normal	0.05			
	PT2_70	IonTorrent_S5XL	CTC			Aberrant				
	PT2_71	IonTorrent_S5XL	CTC			Normal				
PT3	PT3_7	Illumina_MiSeq	CTM	Whole	3	aberrant	0.14	0.248	0	0.555
	PT3_9	IonTorrent_S5XL	CTM	Whole	4	aberrant	0.07	0.082	0	0.386
	PT3_36	Illumina_MiSeq	CTM	Whole	4	aberrant	0.16	0.281	0	0.59
	PT3_37	Illumina_MiSeq	CTM	Whole	4	aberrant	0.1	0.165	0	0.471
	PT3_41	IonTorrent_S5XL	CTM	Whole	4	aberrant	0.08	0.113	0	0.418
	PT3_42	IonTorrent_S5XL	CTM	Whole	4	aberrant	0.12	0.209	0	0.516
	PT3_43	IonTorrent_S5XL	CTM	Whole	4	aberrant	0.09	0.14	0	0.446
	PT3_156	Illumina_MiSeq	CTM	Whole	4	aberrant	0.27	0.424	0.114	0.739
	PT3_161	Illumina_MiSeq	CTM	Whole	4	aberrant	0.09	0.14	0	0.446
	PT3_162	Illumina_MiSeq	CTM	Whole	4	aberrant	0.12	0.209	0	0.516
	PT3_164	Illumina_MiSeq	CTM	Whole	4	aberrant	0.14	0.248	0	0.555
	PT3_44	IonTorrent_S5XL	CTC			aberrant				
PT4	PT4_13	IonTorrent_S5XL	CTM	Whole	4	aberrant	0.28	0.435	0.126	0.751
	PT4_15	IonTorrent_S5XL	CTM	Whole	4	aberrant	0.09	0.14	0	0.446
	PT4_20	IonTorrent_S5XL	CTM	Whole	4	aberrant	0.12	0.209	0	0.516
	PT4_27	IonTorrent_S5XL	CTM	Whole	4	unclear	0.2			
	PT4_28	IonTorrent_S5XL	CTM	Whole	4	aberrant	0.15	0.265	0	0.573
	PT4_31	IonTorrent_S5XL	CTM	Whole	4	aberrant	0.13	0.229	0	0.536
	PT4_33	IonTorrent_S5XL	CTM	Whole	4	aberrant	0.08	0.113	0	0.418
	PT4_118	Illumina_MiSeq	CTM	Whole	4	unclear	0.09			
	PT4_120	Illumina_MiSeq	CTM	Whole	4	aberrant	0.24	0.39	0.079	0.703
	PT4_123	Illumina_MiSeq	CTM	Whole	4	aberrant	0.16	0.281	0	0.59
	PT4_125	Illumina_MiSeq	CTM	Whole	4	unclear	0.22			
	PT4_127	Illumina_MiSeq	CTM	Whole	4	aberrant	0.11	0.188	0	0.494
	PT4_129	Illumina_MiSeq	CTM	Whole	4	aberrant	0.28	0.435	0.126	0.751
PT5	PT5_49	IonTorrent_S5XL	CTM	Whole	4	aberrant	0.11	0.188	0	0.494
	PT5_52	Illumina_MiSeq	CTM	Divided	4	aberrant	0.11	0.188	0	0.494
	PT5_53	IonTorrent_S5XL	CTM	Divided	4	normal	0.02			
	PT5_55	IonTorrent_S5XL	CTM	Whole	4	aberrant	0.08	0.113	0	0.418
	PT5_57	Illumina_MiSeq	CTM	Whole	4	aberrant	0.09	0.14	0	0.446
	PT5_58	Illumina_MiSeq	CTM	Whole	4	aberrant	0.09	0.14	0	0.446
	PT5_136	Illumina_MiSeq	CTM	Whole	4	aberrant	0.1	0.165	0	0.471
	PT5_137	Illumina_MiSeq	CTM	Whole	4	aberrant	0.12	0.209	0	0.516
	PT5_138	Illumina_MiSeq	CTM	Divided	3	aberrant	0.19	0.326	0.012	0.636
	PT5_139	Illumina_MiSeq	CTM	Divided	4	aberrant	0.1	0.165	0	0.471
	PT5_141	Illumina_MiSeq	CTM	Whole	3	aberrant	0.11	0.188	0	0.494
	PT5_143	Illumina_MiSeq	CTM	Whole	3	aberrant	0.11	0.188	0	0.494
	PT5_144	Illumina_MiSeq	CTM	Whole	4	aberrant	0.15	0.265	0	0.573
	PT5_145	Illumina_MiSeq	CTM	Whole	4	aberrant	0.23	0.378	0.066	0.691

	PT5_147	Illumina_MiSeq	CTM	Whole	3	aberrant	0.15	0.265	0	0.573
	PT5_148	Illumina_MiSeq	CTM	Whole	3	aberrant	0.08	0.113	0	0.418
	PT5_54	IonTorrent_S5XL	CTC			Normal				
	PT5_56	IonTorrent_S5XL	CTC			low_reads_count				
	PT6_62	Illumina_MiSeq	CTM	Divided	4	aberrant	0.32	0.476	0.168	0.794
PT6	PT6_63	Illumina_MiSeq	CTM	Divided	4	aberrant	0.1	0.165	0	0.471
	PT6_65	Illumina_MiSeq	CTM	Whole	4	aberrant	0.08	0.113	0	0.418

Table S3. Yields and quality control scores related to microdissected primary tumor samples.

Patient	# of cells Post-dissociation	# of cells to be stained	# of cells Post-staining	QC Score	# cells incubated in DEPArray™ buffer	Incubation time in DEPArray™ Buffer	# of cells loaded on the cartridge
1	3331500	500000	409200	0.194563278	80000	20	24113
2	2977500	500000	568800	0.206834447	80000	41 h	24462
3	3877500	500000	885600	0.301659404	80000	70,25 h	24570
4	3457500	500000	487200	0.491419706	80000	26	14085
5	1162000	500000	222600	0.430408745	80000	31	12060
6	2554500	500000	378000	0.182915963	80000	34,25 h	24852

Table S4A. Top (>70%) shared amplifications between Tumor tissue samples.

Chr	start	end	width	name	Freq
chr8	102000001	103000000	1000000	q22.3	1
chr8	103000001	104000000	1000000	q22.3	1
chr8	104000001	106000000	2000000	q22.3	1
chr8	106000001	107000000	1000000	q22.3,q23.1	1
chr8	107000001	108000000	1000000	q23.1	1
chr8	108000001	110000000	2000000	q23.1	1
chr8	110000001	114000000	4000000	q23.1,q23.2,q23.3	1
chr8	114000001	115000000	1000000	q23.3	1
chr8	115000001	118000000	3000000	q23.3,q24.11	1
chr8	118000001	121000000	3000000	q24.11,q24.12	1
chr8	121000001	123000000	2000000	q24.12,q24.13	1
chr8	123000001	125000000	2000000	q24.13	1
chr8	125000001	126000000	1000000	q24.13	1
chr8	126000001	132000000	6000000	q24.13,q24.21,q24.22	1
chr8	54000001	58000000	4000000	q11.23,q12.1	1
chr8	58000001	61000000	3000000	q12.1	1
chr8	61000001	67000000	6000000	q12.1,q12.2,q12.3,q13.1	1
chr8	67000001	74000000	7000000	q13.1,q13.2,q13.3,q21.11	1
chr8	74000001	77000000	3000000	q21.11	1
chr8	77000001	79000000	2000000	q21.11,q21.12	1
chr8	79000001	86000000	7000000	q21.2,q21.12,q21.13	1
chr8	87000001	96000000	9000000	q21.3,q22.1	1
chr8	96000001	102000000	6000000	q22.1,q22.2,q22.3	1
chr8	132000001	145000000	13000000	q24.3,q24.22,q24.23	0.91
chr8	49000001	54000000	5000000	q11.21,q11.22,q11.23	0.91
chr17	48000001	50000000	2000000	q21.33	0.82
chr17	53000001	56000000	3000000	q22	0.82
chr17	56000001	66000000	10000000	q22,q23.1,q23.2,q23.3,q24.1,q24.2	0.82
chr8	47000001	48000000	1000000	q11.1	0.82
chr8	48000001	49000000	1000000	q11.1,q11.21	0.82
chr8	86000001	87000000	1000000	q21.2,q21.3	0.82
chr17	46000001	48000000	2000000	q21.32,q21.33	0.73
chr20	44000001	51000000	7000000	q13.2,q13.12,q13.13	0.73
chr20	51000001	54000000	3000000	q13.2	0.73
chr20	54000001	61000000	7000000	q13.2,q13.31,q13.32,q13.33	0.73

Table S4B. Top (>70%) shared deletions between Tumor tissues samples.

Chr	start	end	width	name	Freq
chr11	103000001	134000000	31000000	q22.3,q23.1,q23.2,q23.3,q24.1,q24.2,q24.3,q25	0.82
chr16	72000001	76000000	4000000	q22.2,q22.3,q23.1	0.82
chr16	76000001	77000000	1000000	q23.1	0.82

chr16	77000001	79000000	2000000	q23.1	0.82
chr16	79000001	81000000	2000000	q23.1,q23.2	0.82
chr16	81000001	82000000	1000000	q23.2,q23.3	0.82
chr16	82000001	90000000	8000000	q23.3,q24.1,q24.2,q24.3	0.82
chr11	92000001	99000000	7000000	q14.3,q21,q22.1	0.73
chr11	99000001	103000000	4000000	q22.1,q22.2,q22.3	0.73

Table S4C. Top (>50%) shared amplifications between CTMs.

Chr	start	end	width	name	Freq
chr20	41000001	44000000	3000000	q12,q13.11,q13.12	0.66
chr20	44000001	51000000	7000000	q13.2,q13.12,q13.13	0.66
chr20	51000001	54000000	3000000	q13.2	0.66
chr20	54000001	61000000	7000000	q13.2,q13.31,q13.32,q13.33	0.66
chr20	10000001	13000000	3000000	p12.1,p12.2	0.64
chr20	13000001	14000000	1000000	p12.1	0.64
chr20	14000001	25000000	11000000	p11.21,p11.22,p11.23,p12.1	0.64
chr20	25000001	30000000	5000000	p11.1,p11.21,q11.1,q11.21	0.64
chr20	30000001	41000000	11000000	q11.21,q11.22,q11.23,q12	0.64
chr20	1000001	10000000	9000000	p12.2,p12.3,p13	0.62
chr16	81000001	82000000	1000000	q23.2,q23.3	0.58
chr16	82000001	90000000	8000000	q23.3,q24.1,q24.2,q24.3	0.58
chr16	79000001	81000000	2000000	q23.1,q23.2	0.57
chr8	126000001	132000000	6000000	q24.13,q24.21,q24.22	0.55
chr8	132000001	145000000	13000000	q24.3,q24.22,q24.23	0.55
chr8	125000001	126000000	1000000	q24.13	0.53

Table S4D. Top (>50%) shared deletions between CTMs.

Chr	start	end	width	name	Freq
chr17	56000001	66000000	10000000	q22,q23.1,q23.2,q23.3,q24.1,q24.2	0.66
chr17	66000001	68000000	2000000	q24.2,q24.3	0.66
chr17	1000001	15000000	14000000	p12,p13.1,p13.2,p13.3	0.64
chr17	15000001	17000000	2000000	p11.2,p12	0.64
chr17	27000001	44000000	17000000	q11.2,q12,q21.1,q21.2,q21.31	0.64
chr17	44000001	45000000	1000000	q21.31,q21.32	0.64
chr17	45000001	46000000	1000000	q21.32	0.64
chr17	46000001	48000000	2000000	q21.32,q21.33	0.64
chr17	48000001	50000000	2000000	q21.33	0.64
chr17	50000001	53000000	3000000	q21.33,q22	0.64
chr17	53000001	56000000	3000000	q22	0.64
chr17	68000001	69000000	1000000	q24.3	0.64
chr17	17000001	18000000	1000000	p11.2	0.62
chr17	18000001	26000000	8000000	p11.1,p11.2,q11.1,q11.2	0.62
chr17	26000001	27000000	1000000	q11.2	0.62
chr17	69000001	70000000	1000000	q24.3	0.6
chr17	70000001	72000000	2000000	q24.3,q25.1	0.58
chr17	72000001	73000000	1000000	q25.1	0.57
chr17	73000001	75000000	2000000	q25.1,q25.2	0.57
chr17	75000001	81000000	6000000	q25.2,q25.3	0.57

Table S5A: Genomic regions associated with private alterations of CTMs.

seqnames	start	end	width	strand	gene_id	Symbol	Cytoband
chr2	161993466	162092683	99218	+	10010	TANK	q24.2
chr2	203156040	203156151	112	+	100113392	SNORD11B	q33.1
chr2	176032361	176032437	77	-	100126350	MIR933	q31.1
chr2	219866937	219880444	13508	+	100129175	LOC100129175	q35
chr2	214141277	214148929	7653	-	100130451	LOC100130451	q34
chr2	197565359	197577736	12378	-	100130452	LOC100130452	q33.1
chr2	178148242	178257419	109178	-	100130691	LOC100130691	q31.2
chr2	191371619	191399468	27850	-	100131211	NEMP2	q32.2
chr2	175199821	175202268	2448	+	100131390	SP9	q31.1
chr2	111878491	111926022	47532	+	10018	BCL2L11	q13

chr2	104995308	105024790	29483	-	100287010	LOC100287010	q12.1
chr2	109743784	109746575	2792	-	100287216	SH3RF3-AS1	q12.3
chr2	177465708	177465780	73	-	100302142	MIR1246	q31.1
chr2	179246805	179541009	294205	+	100302152	MIR548N	q31.2
chr2	180725563	180725635	73	-	100302172	MIR1258	q31.3
chr2	189842818	189842886	69	+	100302219	MIR1245A	q32.2
chr2	213290987	213291084	98	-	100313771	MIR548F2	q34
chr2	206980297	206981296	1000	-	100329109	GCSHP3	q33.3
chr2	165544153	165544287	135	-	100337591	SNORA70F	q24.3
chr2	178120673	178120738	66	-	100422824	MIR3128	q31.2
chr2	109757946	109758044	99	-	100422863	MIR4265	q12.3
chr2	189997762	189997837	76	-	100422908	MIR3129	q32.2
chr2	219923410	219923472	63	-	100422957	MIR3131	q35
chr2	220771223	220771286	64	-	100422959	MIR4268	q35
chr2	207647958	207648032	75	-	100422993	MIR3130-1	q33.3
chr2	110827538	110827619	82	-	100422994	MIR4267	q13
chr2	207647958	207648032	75	+	100423002	MIR3130-2	q33.3
chr2	109930027	109930081	55	-	100423027	MIR4266	q12.3
chr2	207974711	207974797	87	-	100423036	MIR2355	q33.3
chr2	220413795	220413869	75	-	100423039	MIR3132	q35
chr2	114735246	114737561	2316	-	100499194	LOC100499194	q14.1
chr2	189860356	189860418	63	+	100500837	MIR3606	q32.2
chr2	171627604	171634757	7154	+	100505695	NA	q31.1
chr2	166713986	166728451	14466	-	100506124	LOC100506124	q24.3
chr2	166790367	166804831	14465	+	100506134	TTC21B-AS1	q24.3
chr2	105421883	105467934	46052	-	100506421	PANTR1	q12.1
chr2	176999569	177001826	2258	-	100506783	HOXD-AS2	q31.1
chr2	179387554	179484944	97391	+	100506866	TTN-AS1	q31.2
chr2	201577028	201599900	22873	-	100507140	LINC01792	q33.1
chr2	111003215	111024135	20921	+	100507334	LOC100507334	q13
chr2	208983853	209021486	37634	+	100507443	LOC100507443	q33.3,q34
chr2	209119957	209120918	962	+	100507475	IDH1-AS1	q34
chr2	160625139	160761267	136129	-	100526664	LY75-CD302	q24.2
chr2	170550964	170608396	57433	+	100526832	PHOSPHO2-KLHL23	q31.1
chr2	198364721	198418423	53703	+	100529241	HSPE1-MOB4	q33.1
chr2	220074488	220085174	10687	-	10058	ABCB6	q35
chr2	103048749	103048826	78	+	100616157	MIR4772	q12.1
chr2	114478867	114478945	79	-	100616208	MIR4782	q14.1
chr2	182170320	182170379	60	-	100616213	MIR4437	q31.3
chr2	213790981	213791060	80	+	100616267	MIR4776-1	q34
chr2	189842820	189842887	68	-	100616324	MIR1245B	q32.2
chr2	169439453	169439528	76	+	100616356	MIR4774	q24.3
chr2	208619531	208619605	75	+	100616361	MIR4775	q33.3
chr2	161264321	161264393	73	-	100616364	MIR4785	q24.2
chr2	213790981	213791060	80	-	100616472	MIR4776-2	q34
chr2	202937978	203061886	123909	+	100652824	KIAA2012	q33.1
chr2	101925912	101925996	85	+	100847007	MIR5696	q11.2
chr2	169628461	169642939	14479	-	100861402	CERS6-AS1	q24.3
chr2	215374906	215401613	26708	+	100885780	VWC2L-IT1	q35
chr2	114647511	114719129	71619	+	10096	ACTR3	q14.1
chr2	219081817	219119071	37255	+	10109	ARPC2	q35
chr2	190627506	190630282	2777	+	101409258	OSGEPL1-AS1	q32.2
chr2	204193003	204296892	103890	+	10152	ABI2	q33.2
chr2	169921299	169952677	31379	+	10170	DHRS9	q31.1
chr2	99923088	99957165	34078	-	10190	TXNDC9	q11.2
chr2	188206690	188313021	106332	-	10203	CALCRL	q32.1
chr2	162164786	162268228	103443	+	10213	PSMD14	q24.2
chr2	220299700	220358354	58655	+	10290	SPEG	q35
chr2	211295973	211341499	45527	-	10314	LANCL1	q34
chr2	170336006	170382772	46767	+	10324	KLHL41	q31.1
chr2	112656191	112786945	130755	+	10461	MERTK	q13
chr2	181845112	181928150	83039	+	10477	UBE2E3	q31.3
chr2	177134123	177202753	68631	+	10651	MTX2	q31.1
chr2	162272620	162281573	8954	+	10716	TBR1	q24.2

chr2	183789579	183903586	114008	-	10787	NCKAP1	q32.1
chr2	109510927	109605828	94902	-	10913	EDAR	q12.3
chr2	173600525	173917620	317096	+	11069	RAPGEF4	q31.1
chr2	101623690	101767846	144157	-	11138	TBC1D8	q11.2
chr2	114384817	114400975	16159	+	11159	RABL2A	q13,q14.1
chr2	158114340	158167913	53574	+	11227	GALNT5	q24.1
chr2	175664042	175870107	206066	-	1123	CHN1	q31.1
chr2	105858200	105859924	1725	+	11250	GPR45	q12.1
chr2	99235569	99347589	112021	-	11320	MGAT4A	q11.2
chr2	175612323	175629200	16878	-	1134	CHRNA1	q31.1
chr2	220462596	220481173	18578	+	114790	STK11IP	q35
chr2	179059208	179264160	204953	+	114880	OSBPL6	q31.2
chr2	169643049	169721849	78801	+	115677	NOSTRIN	q24.3,q31.1
chr2	189598465	189654831	56367	-	116093	DIRC1	q32.2
chr2	205410516	206484886	1074371	+	117583	PARD3B	q33.3
chr2	114368816	114384715	15900	-	118433	RPL23AP7	q13
chr2	201717732	201729467	11736	-	1195	CLK1	q33.1
chr2	98962618	99015064	52447	+	1261	CNGA3	q11.2
chr2	189839099	189877472	38374	+	1281	COL3A1	q32.2
chr2	189896641	190044605	147965	-	1290	COL5A2	q32.2
chr2	183984750	184026408	41659	+	129401	NUP35	q32.1
chr2	167744997	168116261	371265	+	129446	XIRP2	q24.3
chr2	200793634	200820459	26826	-	129450	TYW5	q33.1
chr2	101086944	101099742	12799	+	129521	NMS	q11.2
chr2	99900701	99921218	20518	-	129530	LYG1	q11.2
chr2	99785726	99797492	11767	-	129531	MITD1	q11.2
chr2	112895962	112945791	49830	+	129804	FBLN7	q13
chr2	178977182	178994382	17201	+	129831	RBM45	q31.2
chr2	170336006	170363165	27160	+	129880	BBS5	q31.1
chr2	170501935	170550931	48997	-	129881	CCDC173	q31.1
chr2	203637873	203736371	98499	-	130026	ICA1L	q33.2
chr2	198435527	198540584	105058	-	130132	RFTN2	q33.1
chr2	158383279	158485399	102121	-	130399	ACVR1C	q24.1
chr2	170684018	170940639	256622	+	130507	UBR3	q31.1
chr2	201353684	201374792	21109	-	130535	KCTD18	q33.1
chr2	202153147	202222121	68975	-	130540	FLACC1	q33.1
chr2	220408385	220415317	6933	+	130612	TMEM198	q35
chr2	220071506	220074373	2868	+	130617	ZFAND2B	q35
chr2	207804278	207834198	29921	+	130749	CPO	q33.3
chr2	207598942	207630050	31109	-	130752	MDH1B	q33.3
chr2	103353394	103434138	80745	+	130827	TMEM182	q12.1
chr2	159027869	159313265	285397	-	130940	CCDC148	q24.1
chr2	211342406	211543831	201426	+	1373	CPS1	q34
chr2	208394616	208470284	75669	+	1385	CREB1	q33.3
chr2	175936978	176032934	95957	-	1386	ATF2	q31.1
chr2	219128852	219134893	6042	-	14	AAMP	q35
chr2	171034655	171511674	477020	+	140469	MYO3B	q31.1
chr2	219854912	219858127	3216	-	1412	CRYBA2	q35
chr2	209025464	209028297	2834	-	1418	CRYGA	q34
chr2	209007297	209010877	3581	-	1419	CRYGB	q34
chr2	208992861	208994554	1694	-	1420	CRYGC	q33.3
chr2	208986331	209028297	41967	-	1421	CRYGD	q33.3,q34
chr2	183943287	183964722	21436	+	142679	DUSP19	q32.1
chr2	204732511	204738683	6173	+	1493	CTLA4	q33.2
chr2	113239743	113290222	50480	+	150465	TTL	q13
chr2	113495444	113522254	26811	-	150468	CKAP2L	q13
chr2	114195268	114253781	58514	+	150472	CBWD2	q13
chr2	200332821	200337481	4661	+	150538	SATB2-AS1	q33.1
chr2	105050805	105129215	78411	+	150568	LINC01102	q12.1
chr2	99758185	99767928	9744	+	150590	C2orf15	q11.2
chr2	190539219	190611376	72158	+	150709	ANKAR	q32.2
chr2	178414881	178417524	2644	-	150737	TTC30B	q31.2
chr2	203499901	203634480	134580	+	150864	FAM117B	q33.2
chr2	217081612	217084915	3304	+	150967	LINC01963	q35

chr2	111132686	111142113	9428	-	151009	LINC01106	q13
chr2	110300374	110371783	71410	-	151011	SEPTIN10	q13
chr2	210885435	211036051	150617	-	151050	KANSL1L	q34
chr2	187692207	187713897	21691	-	151112	ZSWIM2	q32.1
chr2	180306711	180726232	419522	-	151126	ZNF385B	q31.2,q31.3
chr2	208446077	208490065	43989	-	151194	METTL21A	q33.3
chr2	208576264	208620896	44633	+	151195	CCNYL1	q33.3
chr2	170590356	170608396	18041	+	151230	KLHL23	q31.1
chr2	108938694	108970254	31561	+	151234	SULT1C2P1	q12.3
chr2	182850551	182996109	145559	+	151242	PPP1R1C	q31.3
chr2	201390865	201448818	57954	+	151246	SGO2	q33.1
chr2	202352144	202483905	131762	-	151254	C2CD6	q33.1
chr2	165754709	165812035	57327	-	151258	SLC38A11	q24.3
chr2	220026181	220034817	8637	-	151295	SLC23A3	q35
chr2	219841006	219842644	1639	+	151300	LINC00608	q35
chr2	219125738	219128582	2845	+	151306	GPBAR1	q35
chr2	160092304	160143236	50933	-	151525	WDSUB1	q24.2
chr2	158851691	158992666	140976	+	151531	UPP2	q24.1
chr2	175296300	175351816	55517	-	151556	GPR155	q31.1
chr2	219646472	219680016	33545	+	1593	CYP27A1	q35
chr2	100889753	100939195	49443	-	164832	LONRF2	q11.2
chr2	109403219	109492847	89629	+	165055	CCDC138	q12.3
chr2	187558789	187628512	69724	+	165215	FAM171B	q32.1
chr2	119913819	119916471	2653	-	165257	C1QL2	q14.2
chr2	220283099	220291461	8363	+	1674	DES	q35
chr2	172950208	172954401	4194	+	1745	DLX1	q31.1
chr2	172964166	172967478	3313	-	1746	DLX2	q31.1
chr2	172543919	172606668	62750	+	1781	DYNC1I2	q31.1
chr2	162848755	162931052	82298	-	1803	DPP4	q24.2
chr2	207024318	207027653	3336	+	1933	EEF1B2	q33.3
chr2	114256661	114258727	2067	+	200350	FOXD4L1	q13
chr2	101964816	102003965	39150	-	200407	CREG2	q11.2
chr2	209130991	209223475	92485	+	200576	PIKFYVE	q34
chr2	207507142	207514173	7032	+	200726	FAM237A	q33.3
chr2	119599747	119605759	6013	-	2019	EN1	q14.2
chr2	222282747	222437010	154264	-	2043	EPHA4	q36.1
chr2	110969106	110980516	11411	-	205251	MTLN	q13
chr2	200775979	200792996	17018	+	205327	C2orf69	q33.1
chr2	212240442	213403352	1162911	-	2066	ERBB4	q34
chr2	163027200	163100045	72846	-	2191	FAP	q24.2
chr2	178077422	178088685	11264	+	220988	HNRNPA3	q31.2
chr2	105977283	106055230	77948	-	2274	FHL2	q12.1,q12.2
chr2	213864411	214016333	151923	-	22807	IKZF2	q34
chr2	165541258	165698678	157421	-	22837	COBLL1	q24.3
chr2	207630112	207660911	30800	+	22868	FASTKD2	q33.3
chr2	160797260	160919126	121867	-	22925	PLA2R1	q24.2
chr2	200134223	200335989	201767	-	23314	SATB2	q33.1
chr2	216225179	216300791	75613	-	2335	FN1	q35
chr2	220415450	220436268	20819	-	23363	OBSL1	q35
chr2	198256698	198299771	43074	-	23451	SF3B1	q33.1
chr2	220238180	220264729	26550	-	23549	DNPEP	q35
chr2	113931560	113960677	29118	+	23550	PSD4	q13
chr2	192813772	193059644	245873	-	23671	TMEFF2	q32.3
chr2	183698005	183731498	33494	-	2487	FRZB	q32.1
chr2	169312759	169631644	318886	+	253782	CERS6	q24.3
chr2	172864804	172945587	80784	+	254042	METAP1D	q31.1
chr2	99858711	99871570	12860	-	254773	LYG2	q11.2
chr2	219867568	219906273	38706	-	255101	CFAP65	q35
chr2	171673200	171717659	44460	+	2571	GAD1	q31.1
chr2	163200583	163219148	18566	+	25801	GCA	q24.2
chr2	198380295	198418423	38129	+	25843	MOB4	q33.1
chr2	166604313	166651169	46857	-	2591	GALNT3	q24.3
chr2	219135115	219211516	76402	+	25953	PNKD	q35
chr2	99225042	99234977	9936	+	25972	UNC50	q11.2

chr2	171784948	171823643	38696	+	26003	GORASP2	q31.1
chr2	201170604	201346986	176383	+	26010	SPATS2L	q33.1
chr2	215796266	216003151	206886	-	26154	ABCA12	q35
chr2	191069360	191184771	115412	-	26275	HIBCH	q32.2
chr2	162999379	163008914	9536	-	2641	GCG	q24.2
chr2	113816215	113822320	6106	+	26525	IL36RN	q13
chr2	190920426	190927455	7030	-	2660	MSTN	q32.2
chr2	207026605	207026674	70	+	26798	SNORD51	q33.3
chr2	220036619	220042732	6114	-	27013	CNPPD1	q35
chr2	219536749	219567440	30692	+	27148	STK36	q35
chr2	113779668	113810440	30773	-	27177	IL36B	q13
chr2	113670548	113676458	5911	+	27178	IL37	q13
chr2	113763449	113765621	2173	+	27179	IL36A	q13
chr2	108994421	109004270	9850	+	27233	SULT1C4	q12.3
chr2	168810530	169104105	293576	-	27347	STK39	q24.3
chr2	191745547	191830270	84724	+	2744	GLS	q32.2
chr2	157291965	157470247	178283	+	2820	GPD2	q24.1
chr2	207040042	207082771	42730	-	2825	GPR1	q33.3
chr2	197504356	197597530	93175	+	284992	CCDC150	q33.1
chr2	101887684	101925178	37495	-	284996	RNF149	q11.2
chr2	105363095	105374177	11083	-	284998	LINC01114	q12.1
chr2	106209554	106227016	17463	-	285000	LOC285000	q12.2
chr2	179694484	179914786	220303	-	285025	CCDC141	q31.2
chr2	175190755	175195370	4616	+	285084	LINC01305	q31.1
chr2	162355578	162364413	8836	-	285116	AHCTF1P1	q24.2
chr2	201838441	201936392	97952	-	285172	FAM126B	q33.1
chr2	210636717	210864024	227308	+	285175	UNC80	q34
chr2	218899657	218955304	55648	+	285180	RUFY4	q35
chr2	106998570	107007851	9282	+	285189	PLGLA	q12.2
chr2	108443388	108509000	65613	+	285190	RGPD4	q12.3
chr2	165349323	165478360	129038	-	2888	GRB14	q24.3
chr2	211482295	211484599	2305	+	29034	CPS1-IT1	q34
chr2	170668268	170681420	13153	-	29081	METTL5	q31.1
chr2	174937175	175113365	176191	-	29789	OLA1	q31.1
chr2	204801471	204826298	24828	+	29851	ICOS	q33.2
chr2	220363587	220371718	8132	+	29926	GMPPA	q35
chr2	160175490	160568946	393457	-	29994	BAZ2B	q24.2
chr2	190425316	190448484	23169	-	30061	SLC40A1	q32.2
chr2	201450731	201536217	85487	+	316	AOX1	q33.1
chr2	177053307	177055635	2329	+	3231	HOXD1	q31.1
chr2	177028805	177037826	9022	+	3232	HOXD3	q31.1
chr2	177016113	177017949	1837	+	3233	HOXD4	q31.1
chr2	176994422	176997423	3002	+	3234	HOXD8	q31.1
chr2	176987413	176989645	2233	+	3235	HOXD9	q31.1
chr2	176981492	176984670	3179	+	3236	HOXD10	q31.1
chr2	176969250	176974316	5067	+	3237	HOXD11	q31.1
chr2	176964530	176965488	959	+	3238	HOXD12	q31.1
chr2	176957532	176960666	3135	+	3239	HOXD13	q31.1
chr2	211052716	211090215	37500	-	33	ACADL	q34
chr2	220144040	220151622	7583	+	3300	DNAJB2	q35
chr2	198351308	198364998	13691	-	3329	HSPD1	q33.1
chr2	198364721	198368187	3467	+	3336	HSPE1	q33.1
chr2	174062441	174146764	84324	-	339751	MAP3K20-AS1	q31.1
chr2	209100953	209119806	18854	-	3417	IDH1	q34
chr2	99410309	99552684	142376	-	343990	KIAA1211L	q11.2
chr2	176944835	176948690	3856	-	344191	EVX2	q31.1
chr2	201560446	201658941	98496	+	344454	AOX2P	q33.1
chr2	109745997	110262207	516211	+	344558	SH3RF3	q12.3,q13
chr2	217498127	217529158	31032	+	3485	IGFBP2	q35
chr2	200625259	200715896	90638	-	348751	FTCDNL1	q33.1
chr2	217536828	217560272	23445	-	3488	IGFBP5	q35
chr2	219919142	219925238	6097	-	3549	IHH	q35
chr2	113531492	113542971	11480	-	3552	IL1A	q13
chr2	113587337	113594356	7020	-	3553	IL1B	q13

chr2	102686836	102796334	109499	+	3554	IL1R1	q11.2,q12.1
chr2	113856937	113891593	34657	+	3557	IL1RN	q13
chr2	219027568	219031716	4149	-	3577	CXCR1	q35
chr2	218990013	219001976	11964	+	3579	CXCR2	q35
chr2	218923878	218926013	2136	-	3580	CXCR2P1	q35
chr2	220436954	220440435	3482	+	3623	INHA	q35
chr2	191208196	191236391	28196	+	3628	INPP1	q32.2
chr2	99061321	99207496	146176	+	3631	INPP4A	q11.2
chr2	173292082	173371181	79100	+	3655	ITGA6	q31.1
chr2	182321619	182402468	80850	+	3676	ITGA4	q31.3
chr2	187454790	187545629	90840	+	3685	ITGAV	q32.1
chr2	160958233	161128399	170167	-	3694	ITGB6	q24.2
chr2	114341230	114356613	15384	+	375260	WASH2P	q13
chr2	177494309	177502302	7994	-	375295	LINC01116	q31.1
chr2	182401401	182545392	143992	-	375298	CERKL	q31.3
chr2	219221579	219232817	11239	+	375307	CATIP	q35
chr2	113033178	113097640	64463	+	376940	ZC3H6	q13
chr2	103089762	103150431	60670	+	389015	SLC9A4	q12.1
chr2	115901625	115918920	17296	-	389023	DPP10-AS1	q14.1
chr2	171571857	171574498	2642	+	389058	SP5	q31.1
chr2	208686012	208890284	204273	-	389072	PLEKHM3	q33.3
chr2	209030071	209054773	24703	-	389073	C2orf80	q34
chr2	220192131	220197899	5769	-	389075	RESP18	q35
chr2	100163716	100759037	595322	-	3899	AFF3	q11.2
chr2	207516345	207583120	66776	-	391475	DYTN	q33.3
chr2	109150811	109303702	152892	+	3987	LIMS1	q12.3
chr2	113399407	113401757	2351	-	400999	FLJ42351	q13
chr2	177037924	177053686	15763	-	401022	HAGLR	q31.1
chr2	186603355	186698016	94662	+	401024	FSIP2	q32.1
chr2	197669139	197675000	5862	-	401027	C2orf66	q33.1
chr2	215276461	215440653	164193	+	402117	VWC2L	q34,q35
chr2	169983619	170219122	235504	-	4036	LRP2	q31.1
chr2	160659868	160761267	101400	-	4065	LY75	q24.2
chr2	177015031	177015140	110	+	406903	MIR10B	q31.1
chr2	220158833	220158922	90	-	406944	MIR153-1	q35
chr2	219267369	219267445	77	+	407017	MIR26B	q35
chr2	210288771	210598834	310064	+	4133	MAP2	q34
chr2	114737146	114764887	27742	+	440900	LINC01191	q14.1
chr2	171568949	171571077	2129	-	440925	LINC01124	q31.1
chr2	108863651	108881807	18157	+	442038	SULT1C3	q12.3
chr2	192110107	192290115	180009	+	4430	MYO1B	q32.3
chr2	211154868	211179895	25028	-	4632	MYL1	q34
chr2	191513848	191557492	43645	+	4664	NAB1	q32.2
chr2	201936462	201950473	14012	+	4709	NDUFB3	q33.1
chr2	216176679	216214496	37818	+	471	ATIC	q35
chr2	206987803	207024243	36441	-	4719	NDUFS1	q33.3
chr2	182540833	182545392	4560	-	4760	NEUROD1	q31.3
chr2	178095031	178257419	162389	-	4780	NFE2L2	q31.2
chr2	101436613	101613287	176675	+	4862	NPAS2	q11.2
chr2	110880914	110962639	81726	-	4867	NPHP1	q13
chr2	157180944	157189287	8344	-	4929	NR4A2	q24.1
chr2	99215786	99224981	9196	-	493753	COA5	q11.2
chr2	170550964	170558218	7255	+	493911	PHOSPHO2	q31.1
chr2	219866367	219866430	64	-	494324	MIR375	q35
chr2	179316163	179326110	9948	+	494513	PJVK	q31.2
chr2	201774894	201828424	53531	-	4999	ORC2	q33.1
chr2	217277137	217347774	70638	+	50485	SMARCAL1	q35
chr2	178487977	178973066	485090	-	50940	PDE11A	q31.2
chr2	118846050	118867597	21548	+	51141	INSIG2	q14.2
chr2	99758185	99939196	181012	+	51263	MRPL30	q11.2
chr2	183004762	183387572	382811	-	5136	PDE1A	q32.1
chr2	189156396	189460652	304257	+	51454	GULP1	q32.1,q32.2
chr2	100016938	100106480	89543	-	51455	REV1	q11.2
chr2	99771418	99779613	8196	+	51601	LIPT1	q11.2

chr2	203130515	203168384	37870	+	51602	NOP58	q33.1
chr2	173420101	173489823	69723	+	5163	PDK1	q31.1
chr2	179328391	179343355	14965	-	51661	FKBP7	q31.2
chr2	173940565	174132737	192173	+	51776	MAP3K20	q31.1
chr2	176040986	176046490	5505	-	518	ATP5MC3	q31.1
chr2	198669426	199014608	345183	+	5334	PLCL1	q33.1
chr2	219687106	219696512	9407	-	53632	PRKAG3	q35
chr2	190648811	190742355	93545	+	5378	PMS1	q32.2
chr2	201735679	201753849	18171	-	53938	PPIL3	q33.1
chr2	111965359	112252692	287334	-	541471	MIR4435-2HG	q13
chr2	183580768	183644750	63983	+	54431	DNAJC10	q32.1
chr2	118673054	118771739	98686	-	54520	CCDC93	q14.1
chr2	190526125	190535557	9433	+	54529	ASNSD1	q32.2
chr2	105471969	105473471	1503	+	5455	POU3F3	q12.1
chr2	219845809	219850379	4571	-	54738	FEV	q35
chr2	191273081	191367041	93961	+	54842	MFSD6	q32.2
chr2	206858445	206950906	92462	-	54891	INO80D	q33.3
chr2	164464118	164592513	128396	-	55137	FIGN	q24.3
chr2	220094479	220101391	6913	+	55139	ANKZF1	q35
chr2	119981384	120023227	41844	+	55240	STEAP3	q14.2
chr2	111490150	111875799	385650	+	55289	ACOXL	q13
chr2	159023162	159092681	69520	+	554201	CCDC148-AS1	q24.1
chr2	202316392	202345574	29183	+	55437	STRADB	q33.1
chr2	220378892	220403494	24603	+	55515	ASIC4	q35
chr2	101869345	101886778	17434	+	55571	CNOT11	q11.2
chr2	216807314	216878346	71033	-	55686	MREG	q35
chr2	203745323	203776949	31627	-	55759	WDR12	q33.2
chr2	216861029	216946539	85511	-	55825	PECR	q35
chr2	187350885	187374087	23203	+	55854	ZC3H15	q32.1
chr2	196602427	196933536	331110	-	56171	DNAH7	q32.3
chr2	113735606	113743227	7622	+	56300	IL36G	q13
chr2	196521532	196602426	80895	+	57181	SLC39A10	q32.3
chr2	162480845	162841786	360942	+	57282	SLC4A10	q24.2
chr2	204103164	204170563	67400	+	57404	CYP20A1	q33.2
chr2	169727401	169746944	19544	-	57405	SPC25	q31.1
chr2	209224569	209704818	480250	+	5746	PTH2R	q34
chr2	158175125	158184146	9022	-	57471	ERMN	q24.1
chr2	197063977	197457335	393359	-	57520	HECW2	q32.3,q33.1
chr2	217122585	217236750	114166	-	57574	MARCH4	q35
chr2	115199899	116602326	1402428	+	57628	DPP10	q14.1
chr2	202564986	202645895	80910	-	57679	ALS2	q33.1
chr2	207139523	207179148	39626	+	57683	ZDBF2	q33.3
chr2	219314974	219433084	118111	-	57695	USP37	q35
chr2	180809604	180871840	62237	-	57703	CWC22	q31.3
chr2	169757750	169766510	8761	+	57818	G6PC2	q31.1
chr2	220154345	220174295	19951	-	5798	PTPRN	q35
chr2	215593275	215674428	81154	-	580	BARD1	q35
chr2	219263061	219270664	7604	+	58190	CTDSP1	q35
chr2	202509597	202563417	53821	-	58538	MPP4	q33.1
chr2	109335937	109402267	66331	+	5903	RANBP2	q12.3
chr2	161128662	161350318	221657	-	5937	RBMS1	q24.2
chr2	108602995	108630443	27449	+	60482	SLC5A7	q12.3
chr2	201754050	201768655	14606	+	60491	NIF3L1	q33.1
chr2	210867289	210886984	19696	+	6120	RPE	q34
chr2	101618691	101636155	17465	+	6160	RPL31	q11.2
chr2	217363520	217366188	2669	+	6168	RPL37A	q35
chr2	219524379	219528166	3788	+	617	BCS1L	q35
chr2	207026952	207027083	132	+	619569	SNORA41	q33.3
chr2	166845670	167005642	159973	-	6323	SCN1A	q24.3
chr2	166095912	166248820	152909	+	6326	SCN2A	q24.3
chr2	165944030	166060577	116548	-	6328	SCN3A	q24.3
chr2	167260083	167350717	90635	-	6332	SCN7A	q24.3
chr2	167051697	167232497	180801	-	6335	SCN9A	q24.3
chr2	193614571	193641625	27055	+	64002	PCGEM1	q32.3

chr2	219138917	219157280	18364	-	64114	TMBIM1	q35
chr2	163123589	163175218	51630	-	64135	IFIH1	q24.2
chr2	190611386	190627953	16568	-	64172	OSGEPL1	q32.2
chr2	219528587	219536781	8195	-	64320	RNF25	q35
chr2	216476286	216708259	231974	-	646324	LINC00607	q35
chr2	112525214	112641741	116528	-	64682	ANAPC1	q13
chr2	160569010	160625084	56075	+	64844	MARCH7	q24.2
chr2	192542798	192553248	10451	+	64859	NABP1	q32.3
chr2	105654483	105716418	61936	+	64965	MRPS9	q12.1
chr2	204298539	204400058	101520	-	65059	RAPH1	q33.2
chr2	202655177	202760273	105097	+	65061	CDK15	q33.1
chr2	202484907	202508252	23346	-	65062	TMEM237	q33.1
chr2	203879602	204091101	211500	+	65065	NBEAL1	q33.2
chr2	202005012	202022515	17504	-	65072	CFLAR-AS1	q33.1
chr2	220492292	220506702	14411	+	6508	SLC4A3	q35
chr2	110371911	110376564	4654	+	65124	SOWAHC	q13
chr2	107021136	107084801	63666	-	653489	RGPD3	q12.2
chr2	114334959	114336429	1471	+	654412	FAM138B	q13
chr2	113993104	114024600	31497	+	654433	PAX8-AS1	q13
chr2	103236166	103327809	91644	+	6549	SLC9A2	q12.1
chr2	219246752	219261617	14866	+	6556	SLC11A1	q35
chr2	113403434	113421400	17967	+	6574	SLC20A1	q13
chr2	203241050	203432474	191425	+	659	BMPR2	q33.1,q33.2
chr2	179345199	179369782	24584	+	65977	PLEKHA3	q31.2
chr2	202241930	202316319	74390	-	66008	TRAK2	q33.1
chr2	198591603	198650938	59336	-	66037	BOLL	q33.1
chr2	174771187	174830430	59244	-	6670	SP3	q31.1
chr2	170655322	170668571	13250	+	6741	SSB	q31.1
chr2	182756472	182795464	38993	+	6744	ITPRID2	q31.3
chr2	191833762	191878976	45215	-	6772	STAT1	q32.2
chr2	191894302	192038902	144601	-	6775	STAT4	q32.2,q32.3
chr2	108905095	108926371	21277	+	6819	SULT1C2	q12.3
chr2	203157774	203157857	84	+	692058	SNORD11	q33.1
chr2	203141154	203141241	88	+	692110	SNORD70	q33.1
chr2	101889398	101889511	114	-	692205	SNORD89	q11.2
chr2	189162219	189162315	97	+	693146	MIR561	q32.1
chr2	111395409	111435684	40276	-	699	BUB1	q13
chr2	188328958	188419219	90262	-	7035	TFPI	q32.1
chr2	217724182	217724782	601	-	7141	TNP1	q35
chr2	218664512	218867718	203207	-	7145	TNS1	q35
chr2	179390717	179672150	281434	-	7273	TTN	q31.2
chr2	220115001	220119330	4330	-	7277	TUBA4A	q35
chr2	113125946	113192062	66117	-	727851	RGPD8	q13
chr2	108439520	108443285	3766	-	729121	RGPD4-AS1	q12.3
chr2	218148746	218621316	472571	-	729582	DIRC3	q35
chr2	102013823	102091165	77343	-	731220	RFX8	q11.2
chr2	203070903	203103322	32420	-	7341	SUMO1	q33.1
chr2	219283838	219314248	30411	+	7429	VIL1	q35
chr2	175424302	175547627	123326	-	7456	WIPF1	q31.1
chr2	219724546	219738954	14409	+	7475	WNT6	q35
chr2	216974020	217071016	96997	+	7520	XRCC5	q35
chr2	219502640	219524355	21716	-	7701	ZNF142	q35
chr2	113973574	114036498	62925	-	7849	PAX8	q13
chr2	102608306	102644884	36579	+	7850	IL1R2	q11.2
chr2	110841447	110874143	32697	-	7851	MALL	q13
chr2	208627310	208634143	6834	-	7855	FZD5	q33.3
chr2	101179418	101193201	13784	+	79031	PDCL3	q11.2
chr2	220082847	220094361	11515	-	79065	ATG9A	q35
chr2	105954013	105961984	7972	+	79074	C2orf49	q12.1
chr2	220042939	220050197	7259	+	79137	RETREG2	q35
chr2	220101503	220110131	8629	-	79411	GLB1L	q35
chr2	200820040	200828847	8808	+	79568	MAIP1	q33.1
chr2	214149103	215275225	1126123	+	79582	SPAG16	q34
chr2	220403669	220408487	4819	-	79586	CHPF	q35

chr2	175260457	175294303	33847	+	79634	SCRN3	q31.1
chr2	170386263	170430424	44162	-	79675	FASTKD1	q31.1
chr2	203776978	203851060	74083	+	79800	CARF	q33.2
chr2	166713986	166810348	96363	-	79809	TTC21B	q24.3
chr2	172173913	172291312	117400	-	79828	METTL8	q31.1
chr2	219940046	220034817	94772	-	79840	NHEJ1	q35
chr2	172378757	172414643	35887	+	79901	CYBRD1	q31.1
chr2	166326157	166545917	219761	+	80034	CSRNP3	q24.3
chr2	197697728	197792519	94792	-	80055	PGAP1	q33.1
chr2	172290727	172341562	50836	+	80067	DCAF17	q31.1
chr2	220117965	220136910	18946	+	80086	TUBA4B	q35
chr2	106709759	106810795	101037	-	80146	UXS1	q12.2
chr2	198318231	198339851	21621	+	80219	COQ10B	q33.1
chr2	114471930	114514400	42471	-	80255	SLC35F5	q14.1
chr2	219745255	219758651	13397	+	80326	WNT10A	q35
chr2	99613724	99771187	157464	-	80705	TSGA10	q11.2
chr2	176790410	176867514	77105	-	80856	LNPK	q31.1
chr2	202899310	202903160	3851	+	8324	FZD7	q33.1
chr2	174219561	174233718	14158	+	83879	CDCA7	q31.1
chr2	202098166	202152434	54269	+	841	CASP8	q33.1
chr2	190306159	190340264	34106	+	84128	WDR75	q32.2
chr2	113299492	113334727	35236	+	84172	POLR1B	q13
chr2	113342036	113346850	4815	+	84269	CHCHD5	q13
chr2	191002486	191068210	65725	+	84281	C2orf88	q32.2
chr2	202047621	202094129	46509	+	843	CASP10	q33.1
chr2	192699032	192712006	12975	-	8436	CAVIN2	q32.3
chr2	106361520	106510730	149211	+	8440	NCK2	q12.2
chr2	106682113	106694609	12497	+	84417	ECRG4	q12.2
chr2	112973439	113012664	39226	-	84524	ZC3H8	q13
chr2	107418056	107503563	85508	-	84620	ST6GAL2	q12.2,q12.3
chr2	113825547	113833427	7881	+	84639	IL1F10	q13
chr2	114356605	114359153	2549	-	84771	DDX11L2	q13
chr2	103333666	103353347	19682	-	84804	MFSD9	q12.1
chr2	219472488	219501909	29422	+	84812	PLCD4	q35
chr2	112812800	112876895	64096	+	84910	TMEM87B	q13
chr2	159313476	159537940	224465	+	8502	PKP4	q24.1
chr2	172778935	172848600	69666	+	8520	HAT1	q31.1
chr2	178257471	178408564	151094	+	8540	AGPS	q31.2
chr2	159825146	160089170	264025	+	85461	TANC1	q24.2
chr2	179296141	179315958	19818	-	8575	PRKRA	q31.2
chr2	220110192	220115059	4868	+	8576	STK16	q35
chr2	172639915	172750816	110902	-	8604	SLC25A12	q31.1
chr2	207938862	208031970	93109	-	8609	KLF7	q33.3
chr2	169779449	169887833	108385	-	8647	ABCB11	q31.1
chr2	119699745	119752236	52492	+	8685	MARCO	q14.2
chr2	168675182	168727366	52185	+	8708	B3GALT1	q24.3
chr2	207308368	207485854	177487	+	8745	ADAM23	q33.3
chr2	103035254	103069024	33771	+	8807	IL18RAP	q12.1
chr2	102803433	102855811	52379	+	8808	IL1RL2	q12.1
chr2	102927962	103015217	87256	+	8809	IL18R1	q12.1
chr2	206547224	206662857	115634	+	8828	NRP2	q33.3
chr2	201980877	202037411	56535	+	8837	CFLAR	q33.1
chr2	118572255	118589953	17699	+	8886	DDX18	q14.1
chr2	219824350	219826877	2528	+	8941	CDK5R2	q35
chr2	158592958	158732374	139417	-	90	ACVR1	q24.1
chr2	163227917	163695257	467341	-	90134	KCNH7	q24.2
chr2	173587918	173600934	13017	-	91149	RAPGEF4-AS1	q31.1
chr2	219433303	219461158	27856	+	9125	CNOT9	q35
chr2	179966419	180129350	162932	-	91404	SESTD1	q31.2
chr2	197831741	198175521	343781	-	91526	ANKRD44	q33.1
chr2	102927962	102968497	40536	+	9173	IL1RL1	q12.1
chr2	185463093	185804214	341122	+	91752	ZNF804A	q32.1
chr2	178479026	178483694	4669	-	92104	TTC30A	q31.2
chr2	159651829	159719471	67643	+	92196	DAPL1	q24.1

chr2	196998307	197041227	42921	-	9262	STK17B	q32.3
chr2	216946589	216967506	20918	+	92691	TMEM169	q35
chr2	198570028	198573114	3087	+	92935	MARS2	q33.1
chr2	197627756	197664492	36737	-	9330	GTF3C3	q33.1
chr2	170440850	170494254	53405	+	9360	PPIG	q31.1
chr2	105883540	105946148	62609	-	9392	TGFBRAP1	q12.1
chr2	204571198	204603636	32439	+	940	CD28	q33.2
chr2	190634993	190649097	14105	-	94101	ORMDL1	q32.2
chr2	102314165	102511152	196988	+	9448	MAP4K4	q11.2
chr2	101008322	101034130	25809	-	9486	CHST10	q11.2
chr2	175212878	175260443	47566	-	9541	CIR1	q31.1
chr2	158271131	158345473	74343	-	9595	CYTIP	q24.1
chr2	109065577	109125854	60278	+	9648	GCC2	q12.3
chr2	219575568	219620138	44571	+	9654	TTLL4	q35
chr2	99953834	100016728	62895	+	9669	EIF5B	q11.2
chr2	201676269	201688569	12301	+	9689	BZW1	q33.1
chr2	171847333	172087824	240492	-	9874	TLK1	q31.1
chr2	160625139	160654766	29628	-	9936	CD302	q24.2

Table S5B. Genomic regions associated with private alterations of CTMs involved in Breast cancer (CancerIndex).

seqnames	gene_biotype	symbol	Cytoband	Frequency_Del	Frequency_Normal	Frequency_Amp
chr2	protein_coding	IL1RL1	q12.1	0.11	0.45	0.43
chr2	protein_coding	FHL2	q12.1,q12.2	0.1	0.46	0.44
chr2	protein_coding	C2orf40	q12.2	0.09	0.45	0.45
chr2	protein_coding	MALL	q13	0.09	0.45	0.45
chr2	protein_coding	BUB1	q13	0.09	0.45	0.45
chr2	protein_coding	TTL	q13	0.09	0.45	0.45
chr2	protein_coding	IL1A	q13	0.09	0.45	0.45
chr2	protein_coding	IL1B	q13	0.09	0.45	0.45
chr2	protein_coding	MARCO	q14.2	0.11	0.45	0.43
chr2	protein_coding	TANK	q24.2	0.13	0.45	0.42
chr2	protein_coding	ITGA6	q31.1	0.15	0.45	0.4
chr2	protein_coding	PDK1	q31.1	0.15	0.45	0.4
chr2	protein_coding	ATF2	q31.1	0.15	0.45	0.4
chr2	protein_coding	HOXD10	q31.1	0.15	0.45	0.4
chr2	protein_coding	NFE2L2	q31.2	0.15	0.45	0.4
chr2	protein_coding	ITGA4	q31.3	0.15	0.45	0.4
chr2	protein_coding	FRZB	q32.1	0.15	0.45	0.4
chr2	protein_coding	TFPI	q32.1	0.15	0.45	0.4
chr2	protein_coding	STAT1	q32.2	0.15	0.45	0.4
chr2	protein_coding	BOLL	q33.1	0.15	0.45	0.4
chr2	protein_coding	FTCDNL1	q33.1	0.15	0.45	0.4
chr2	protein_coding	CASP10	q33.1	0.15	0.45	0.4
chr2	protein_coding	CASP8	q33.1	0.15	0.45	0.4
chr2	protein_coding	SUMO1	q33.1	0.15	0.45	0.4
chr2	protein_coding	BMPR2	q33.1,q33.2	0.15	0.45	0.4
chr2	protein_coding	CTLA4	q33.2	0.15	0.45	0.4
chr2	protein_coding	CREB1	q33.3	0.15	0.45	0.4
chr2	protein_coding	ERBB4	q34	0.15	0.45	0.4
chr2	protein_coding	BARD1	q35	0.15	0.45	0.4
chr2	protein_coding	IGFBP2	q35	0.15	0.45	0.4
chr2	protein_coding	IGFBP5	q35	0.15	0.45	0.4
chr2	protein_coding	CXCR2	q35	0.15	0.45	0.4
chr2	protein_coding	CXCR1	q35	0.15	0.45	0.4

Table S6A. Quality score associated with cell lines raw sequences.

Sample Name	% Dups	% GC	Reads Length	Milion of reads	Sequencing Platform
MDA-MB-361-10-1	14.40%	44%	150 bp	0.8	Illumina_MiSeq
MDA-MB-361-10-2	17.20%	44%	151 bp	1	Illumina_MiSeq
MDA-MB-361-10-3	13.70%	44%	150 bp	0.6	Illumina_MiSeq
MDA-MB-361-100-1	18.10%	44%	150 bp	1.2	Illumina_MiSeq
MDA-MB-361-100-2	17.00%	44%	150 bp	1	Illumina_MiSeq

MDA-MB-361-100-3	19.10%	44%	150 bp	1.2	Illumina_MiSeq
MDA-MB-361-20-1	17.70%	44%	151 bp	1.2	Illumina_MiSeq
MDA-MB-361-20-2	16.50%	44%	150 bp	1	Illumina_MiSeq
MDA-MB-361-20-3	15.80%	44%	150 bp	0.9	Illumina_MiSeq
MDA-MB-361-40-1	15.30%	44%	150 bp	0.9	Illumina_MiSeq
MDA-MB-361-40-2	14.80%	44%	151 bp	0.8	Illumina_MiSeq
MDA-MB-361-40-3	18.40%	44%	151 bp	1.3	Illumina_MiSeq
MDA-MB-361-60-1	17.90%	44%	150 bp	1.2	Illumina_MiSeq
MDA-MB-361-60-2	24.70%	44%	151 bp	1.9	Illumina_MiSeq
MDA-MB-361-60-3	19.10%	44%	150 bp	1.3	Illumina_MiSeq
MDA-MB-361-80-1	15.20%	45%	150 bp	0.8	Illumina_MiSeq
MDA-MB-361-80-2	13.70%	45%	150 bp	0.7	Illumina_MiSeq
MDA-MB-361-80-3	15.60%	45%	151 bp	0.8	Illumina_MiSeq
MDA-MB-453-10-1	10.00%	44%	150 bp	0.4	Illumina_MiSeq
MDA-MB-453-10-2	8.50%	44%	149 bp	0.6	Illumina_MiSeq
MDA-MB-453-10-3	10.80%	44%	149 bp	0.5	Illumina_MiSeq
MDA-MB-453-100-1	14.50%	44%	150 bp	0.7	Illumina_MiSeq
MDA-MB-453-100-2	11.40%	44%	150 bp	0.5	Illumina_MiSeq
MDA-MB-453-100-3	13.60%	44%	149 bp	0.8	Illumina_MiSeq
MDA-MB-453-20-1	12.40%	45%	150 bp	0.7	Illumina_MiSeq
MDA-MB-453-20-2	9.60%	44%	150 bp	0.4	Illumina_MiSeq
MDA-MB-453-20-3	9.80%	44%	150 bp	0.4	Illumina_MiSeq
MDA-MB-453-40-1	9.90%	44%	150 bp	0.4	Illumina_MiSeq
MDA-MB-453-40-2	15.90%	44%	150 bp	1	Illumina_MiSeq
MDA-MB-453-40-3	15.10%	44%	150 bp	1	Illumina_MiSeq
MDA-MB-453-60-1	13.50%	44%	149 bp	0.8	Illumina_MiSeq
MDA-MB-453-60-2	11.70%	44%	149 bp	0.6	Illumina_MiSeq
MDA-MB-453-60-3	17.10%	44%	150 bp	1.2	Illumina_MiSeq
MDA-MB-453-80-1	13.10%	45%	150 bp	0.6	Illumina_MiSeq
MDA-MB-453-80-2	17.50%	45%	150 bp	1.1	Illumina_MiSeq
MDA-MB-453-80-3	17.80%	44%	150 bp	1.2	Illumina_MiSeq
PBL-100-1	10.90%	44%	150 bp	0.5	Illumina_MiSeq
PBL-100-2	10.60%	44%	150 bp	0.4	Illumina_MiSeq

Table S6B. Quality score associated with single and cluster of cells raw sequences.

Sample Name	% Dups	% GC	Length	M Seqs	Sequencing Platform
PT3_7	12.50%	43%	149 bp	1.2	Illumina_MiSeq
PT3_9	12.70%	41%	277 bp	1	IonTorrent_S5XL
PT4_13	11.50%	42%	276 bp	1	IonTorrent_S5XL
PT4_15	10.60%	42%	278 bp	1	IonTorrent_S5XL
PT4_20	12.20%	42%	266 bp	0.9	IonTorrent_S5XL
PT4_27	8.80%	42%	276 bp	0.8	IonTorrent_S5XL
PT4_28	10.00%	42%	263 bp	0.6	IonTorrent_S5XL
PT4_31	8.70%	41%	276 bp	0.7	IonTorrent_S5XL
PT4_33	12.00%	41%	277 bp	1.1	IonTorrent_S5XL
PT3_36	12.50%	44%	150 bp	0.9	Illumina_MiSeq
PT3_37	11.30%	44%	150 bp	0.8	Illumina_MiSeq
PT3_41	10.60%	43%	262 bp	0.8	IonTorrent_S5XL
PT3_42	9.40%	42%	258 bp	0.9	IonTorrent_S5XL
PT3_43	8.90%	42%	265 bp	1.2	IonTorrent_S5XL
PT3_44	12.20%	42%	278 bp	1	IonTorrent_S5XL
PT5_49	8.20%	42%	246 bp	1	IonTorrent_S5XL
PT5_51	9.50%	42%	263 bp	0.6	IonTorrent_S5XL
PT5_53	8.00%	42%	263 bp	1	IonTorrent_S5XL
PT5_54	8.30%	42%	261 bp	0.7	IonTorrent_S5XL
PT5_55	10.00%	42%	269 bp	0.9	IonTorrent_S5XL
PT5_56	10.00%	43%	265 bp	0.3	IonTorrent_S5XL
PT5_57	12.50%	43%	150 bp	1	Illumina_MiSeq
PT5_58	11.60%	43%	150 bp	1	Illumina_MiSeq
PT6_62	9.60%	43%	150 bp	0.6	Illumina_MiSeq
PT6_63	13.20%	44%	150 bp	1	Illumina_MiSeq
PT6_65	9.70%	43%	149 bp	0.8	Illumina_MiSeq
PT2_68	5.70%	42%	248 bp	0.4	IonTorrent_S5XL

PT2_69	6.10%	42%	235 bp	0.7	IonTorrent_S5XL
PT2_70	8.80%	42%	268 bp	1	IonTorrent_S5XL
PT2_71	12.00%	42%	265 bp	0.9	IonTorrent_S5XL
PT2_72	10.20%	41%	261 bp	1.1	IonTorrent_S5XL
PT2_73	8.60%	41%	257 bp	0.9	IonTorrent_S5XL
PT2_74	8.70%	42%	264 bp	0.8	IonTorrent_S5XL
PT2_75	7.90%	41%	260 bp	0.7	IonTorrent_S5XL
PT2_76	8.20%	42%	268 bp	0.8	IonTorrent_S5XL
PT2_77	6.50%	42%	237 bp	0.3	IonTorrent_S5XL
PT2_79	14.70%	42%	265 bp	0.9	IonTorrent_S5XL
PT2_80	8.40%	42%	276 bp	0.6	IonTorrent_S5XL
PT2_81	8.40%	41%	268 bp	0.7	IonTorrent_S5XL
PT2_83	8.70%	41%	257 bp	0.6	IonTorrent_S5XL
PT1_90	8.90%	41%	265 bp	0.7	IonTorrent_S5XL
PT1_93	11.40%	43%	150 bp	0.9	Illumina_MiSeq
PT1_94	11.30%	43%	150 bp	0.9	Illumina_MiSeq
PT1_96	10.00%	43%	150 bp	0.7	Illumina_MiSeq
PT1_98	11.00%	43%	150 bp	0.8	Illumina_MiSeq
PT4_118	11.00%	43%	150 bp	0.9	Illumina_MiSeq
PT4_120	11.00%	44%	150 bp	0.9	Illumina_MiSeq
PT4_123	10.90%	43%	150 bp	0.7	Illumina_MiSeq
PT4_125	10.40%	43%	150 bp	0.7	Illumina_MiSeq
PT4_127	10.60%	43%	150 bp	0.8	Illumina_MiSeq
PT4_129	10.80%	43%	150 bp	0.8	Illumina_MiSeq
PT2_131	5.60%	42%	240 bp	0.5	IonTorrent_S5XL
PT2_133	7.60%	41%	251 bp	0.5	IonTorrent_S5XL
PT2_134	9.90%	41%	254 bp	0.6	IonTorrent_S5XL
PT5_136	12.70%	44%	150 bp	0.9	Illumina_MiSeq
PT5_137	8.90%	43%	151 bp	0.4	Illumina_MiSeq
PT5_138	11.00%	43%	150 bp	0.7	Illumina_MiSeq
PT5_139	11.60%	43%	150 bp	0.8	Illumina_MiSeq
PT5_141	9.60%	43%	150 bp	0.6	Illumina_MiSeq
PT5_143	12.00%	43%	150 bp	0.8	Illumina_MiSeq
PT5_144	10.60%	43%	150 bp	0.8	Illumina_MiSeq
PT5_145	11.90%	43%	150 bp	0.8	Illumina_MiSeq
PT5_147	12.50%	43%	150 bp	0.9	Illumina_MiSeq
PT5_148	9.40%	42%	150 bp	0.5	Illumina_MiSeq
PT3_156	12.00%	43%	150 bp	0.9	Illumina_MiSeq
PT3_161	11.50%	43%	150 bp	0.9	Illumina_MiSeq
PT3_162	11.20%	43%	150 bp	0.8	Illumina_MiSeq
PT3_164	11.00%	43%	150 bp	0.8	Illumina_MiSeq

Table S6C. Quality score associated with tissue cell populations raw sequences.

Sample Name	% Dups	% GC	Reads Length	Milion of reads	Sequencing Platform
PT1_Stromal	14.90%	44%	144 bp	1.8	Illumina_MiSeq
PT1_Tumor1	17.30%	47%	143 bp	1.3	Illumina_MiSeq
PT1_Tumor2	7.40%	40%	146 bp	0.6	Illumina_MiSeq
PT2_Stromal	9.00%	40%	142 bp	0.6	Illumina_MiSeq
PT2_Tumor1	8.80%	42%	144 bp	0.6	Illumina_MiSeq
PT2_Tumor2	6.60%	40%	143 bp	0.5	Illumina_MiSeq
PT3_Stromal	7.60%	41%	146 bp	1.1	Illumina_MiSeq
PT3_Tumor2	10.30%	40%	143 bp	2	Illumina_MiSeq
PT4_Stromal	3.60%	40%	147 bp	0.6	Illumina_MiSeq
PT4_Tumor1	5.20%	41%	146 bp	0.7	Illumina_MiSeq
PT4_Tumor2	5.50%	41%	147 bp	0.8	Illumina_MiSeq
PT5_Stromal	5.10%	40%	147 bp	4.9	Illumina_MiSeq
PT5_Tumor1	8.90%	43%	145 bp	0.6	Illumina_MiSeq
PT5_Tumor2	7.10%	41%	147 bp	2	Illumina_MiSeq
PT6_Stromal	13.60%	43%	144 bp	2.1	Illumina_MiSeq
PT6_Tumor1	14.30%	40%	138 bp	2.5	Illumina_MiSeq
PT6_Tumor2	27.40%	43%	139 bp	2.1	Illumina_MiSeq

Table S7. R packages considered for statistical analysis.

**R packages name**
DescTools
BlandAltmanLeh
nlme
investr
ComplexHeatmap
CNTools
AnnotationHub
